# Genomic Evidence for Dual Introductions, Limited Gene Flow and Niche Preferences in the Invasive Wasp *Vespula germanica* in South Africa

**DOI:** 10.1111/mec.70217

**Published:** 2025-12-22

**Authors:** Damien Gergonne, Ruan Veldtman, Gulu Bekker, Cang Hui, Barbara van Asch

**Affiliations:** ^1^ Centre for Invasion Biology, Department of Mathematical Sciences Stellenbosch University Stellenbosch South Africa; ^2^ Department of Genetics Stellenbosch University Stellenbosch South Africa; ^3^ South African National Biodiversity Institute Kirstenbosch National Botanical Garden Cape Town South Africa; ^4^ Department of Conservation Ecology and Entomology Stellenbosch University Stellenbosch South Africa; ^5^ National Institute for Theoretical and Computational Sciences African Institute for Mathematical Sciences Muizenberg South Africa

**Keywords:** 2b‐RAD sequencing, European wasp, German wasp, population genomics, social wasps

## Abstract

Biological invasions are major drivers of recent biodiversity changes, yet the genetic structure and ecological mechanisms underlying invasion dynamics remain poorly resolved in invasive social insects. In South Africa, the European wasp *Vespula germanica* (Hymenoptera: Vespidae), introduced in the 1970s, has spread remarkably more slowly than in other regions and its geographical distribution in the country remains limited. Although two mitochondrial haplotypes have been reported in South Africa, the species' range expansion and fine‐scale population structure remain poorly understood. Using 2b‐RAD sequencing of 47 colonies across its entire range in South Africa, we identified two main genetic clusters that correspond to previously identified mitochondrial haplotypes, strongly supporting the double introduction scenario. This well‐defined genetic structure is sustained by limited gene flow and possibly by niche preferences. Spatial patterns reveal a dispersal system of short‐range natural movements and human‐mediated jumps, with Stellenbosch acting as a secondary introduction point. Ecological niche preferences appear to maintain a genetic structure through isolation by environment: one group occupies warmer, drier sites, while the other is confined to cooler, wetter microclimates near Cape Town. This pattern reflects ecological adaptation that is likely tied to distinct population origins. As the first genome‐wide study of *V. germanica*, this work illuminates how introduction history and ecological constraints shape invasion dynamics, laying a foundation for understanding the genetic structure and invasion dynamics of the species and for developing predictive management frameworks.

## 1 Introduction

Biological invasions are one of the main drivers of recent changes in biodiversity (Mollot et al. 2017; Seebens et al. 2017). Post‐introduction invasive species establish and expand their geographic ranges through population recruitment and dispersal, leading to diverse invasion dynamics (Hui and Richardson 2017). These dynamics are also contingent upon dispersal mechanisms, habitat conditions and propagule pressure, including introduction history. Studying invasive populations at the genetic level is essential for gaining a better understanding of the dispersal mechanisms of invasive species, a crucial factor in predicting the risk of biological invasions (Bayles et al. 2017; Estoup and Guillemaud 2010; Sakai et al. 2001; Sanders et al. 2001; Seebens et al. 2017).

The European wasp *Vespula germanica* L. (Fabricius) (Hymenoptera: Vespidae) is the most widely distributed invasive social wasp species (De Villiers et al. 2017). Native to the Palaearctic region, *V. germanica* was introduced to the USA, Canada, Chile, Argentina, Iceland, Ascension Island, South Africa, Australia and New Zealand (Akre et al. 1989; Chapman and Bourke 2001; Allsopp and Tribe 2003; Tezcan et al. 2005; van Zyl et al. 2018). The present global distribution is attributed to the feeding plasticity of the species (Farji‐Brener and Corley 1998; Lozada and D'Adamo 2006, 2011), its visual spatial resolution and optical sensitivity (Gutiérrez et al. 2024) and its capacity to thrive in diverse habitats and climates (Bertelsmeier 2021; De Villiers et al. 2017).

Dispersal, defined as the movement of individuals from their natal or breeding habitat to another breeding site, shapes population structure and genetic diversity by affecting gene flow (Ronce 2007; McCauley 2010; Ruf et al. 2011; Clobert 2012). Dispersal of *V. germanica* occurs through both active and passive means. Active dispersal involves the independent movement of reproductive individuals, primarily gynes, driven by their intrinsic mobility rather than environmental or human factors. Studies on invasive populations of *V. germanica* suggest that active dispersal distances are influenced by factors such as overwintering conditions, queen weight before flight and wing surface area. However, these distances are generally short, typically a few hundred meters (Masciocchi et al. 2018; Masciocchi and Corley 2013). During flight, gynes release pheromones to attract males, while males typically show more localised movement patterns, often aggregating near nest sites or mating areas (Moller et al. 1991; Goodisman et al. 2002; Brown et al. 2013; Martínez et al. 2018; Masciocchi et al. 2018, 2020). However, the role of males in the dispersal and gene flow of *V. germanica* is still poorly understood. Passive dispersal occurs when abiotic or human factors extend the travel distances of reproductive individuals or facilitate the translocation of parts of the colony. The transport of overwintered *V. germanica* gynes in human goods such as wood is probably a key factor contributing to their geographical dispersal (Crosland 1991; Spradbery and Maywald 1992; van Zyl et al. 2018). Distinguishing natural from human‐mediated dispersal is challenging in invasive species, but genomic and spatial analyses can provide indirect evidence of their relative roles (Cooke et al. 2025).

Natural and human‐mediated dispersal, combined with the species' adaptability, likely explain why invasions of *V. germanica* are typically rapid and spread across large areas in a short period of time (Clapperton et al. 1989; Crosland 1991; Greene 1991; Tribe 1994; Farji‐Brener and Corley 1998; Goodisman, Matthews, and Crozier 2001). In Argentina, the species expanded its range by 37.2 km per year (Sherpa and Després 2021), spreading over 1000 km southwards in a few decades (Masciocchi and Corley 2013). In New Zealand, it expanded at a rate of 30–47 km per year, invading 80,000 km^2^ in 6 years (Clapperton et al. 1994). In Australia and Tasmania, the rate of expansion was even faster, at 60–70 km and 64 km per year, respectively (Crosland 1991; Spradbery and Maywald 1992). In contrast, since its first observation in South Africa in 1972 (Whitehead and Prins 1975), *V. germanica* has remained confined to the Western Cape Province, the southwest corner of the country, with a geographical range extending only about 150 km from its presumed origin in Cape Town (Veldtman et al. 2012; van Zyl et al. 2018; Davies et al. 2020). The much slower spread of *V. germanica* in South Africa compared with other regions may be due to a combination of environmental, geographical, human and biological factors, as well as founder effects (Sakai et al. 2001; Veldtman et al. 2021), which often results in a reduction in genetic diversity relative to the source population (Sherpa and Després 2021). Founder effects have been widely documented in invasive social‐insect species (Cheng et al. 2016; Husseneder et al. 2012), especially in wasps (Goodisman, Evans, et al. 2001; Arca et al. 2012; Tsuchida et al. 2014; Chau et al. 2015; Takeuchi et al. 2017; Schmack et al. 2019). However, factors underlying the comparatively slower spread of *V. germanica* in South Africa (Veldtman et al. 2021), relative to other invaded regions, are still not well understood.

**FIGURE 1:**
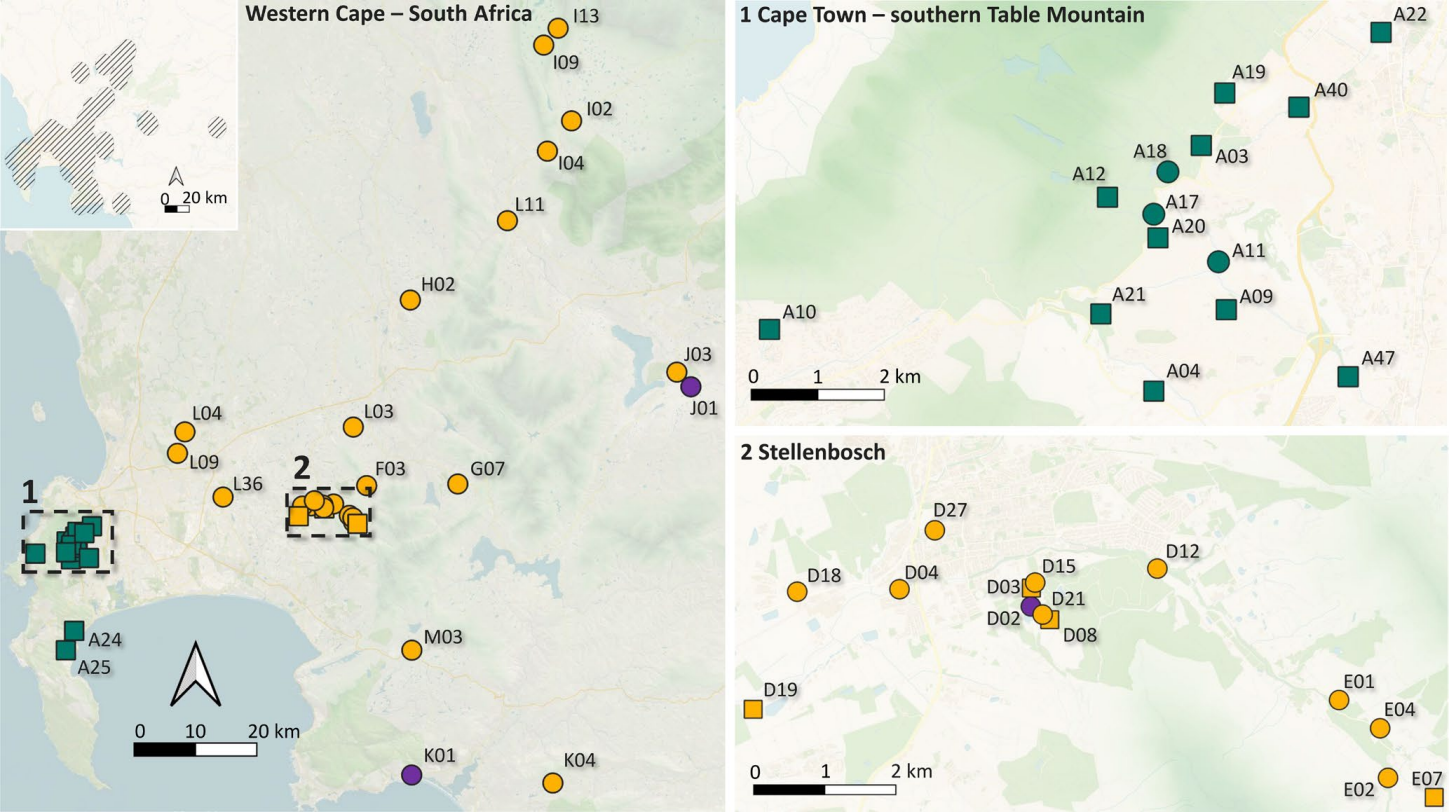
Geographical locations of sampled *Vespula germanica* colonies across its entire distribution range in South Africa and their genetic affiliations. Top left: Current distribution map based on iNaturalist records (September 2025) and our data. Sampling point colours indicate the genetic clusters differentiated based on the nuclear SNP in this study (yellow, P1; green, P2; purple, P3), while sampling point shapes represent mitochondrial haplotypes (circle, H1; square, H2). Colony codes are shown next to corresponding points. Basemap: 2024 Bing Maps, Virtual Earth, Carto Voyager and SRTM GL1 (30 m) elevation data.

The genetic study of invasive populations offers a key to understanding their dispersal mechanisms (Estoup and Guillemaud 2010; Sakai et al. 2001). A recent study based on mitogenomic data revealed the presence of two *V. germanica* haplotypes in South Africa, suggesting two distinct invasion events (van Asch et al. 2022). Haplotype H1 is predominant across the species' range in the country, while the rarer haplotype H2 is primarily found in the Cape Town area (van Asch et al. 2022) (Figure 1). This geographical structure could reflect differences in environmental adaptability, particularly with regards to temperature and humidity, which play a role in the species' overall distribution in South Africa (De Villiers et al. 2017; Veldtman et al. 2021), or limited human transport between Cape Town and other regions.

We investigated the population dynamics behind the spread and establishment of *V. germanica* in South Africa by examining genomic data from 47 colonies using 2b‐RAD sequencing. This study provides insights into how dispersal and environmental factors jointly determine the spread of this invasive species by examining (i) population genomic structure and potential admixture, (ii) the contribution of natural and human‐mediated dispersal and (iii) environmental factors affecting genetic differentiation.

## 2 Materials and Methods

### 2.1 Sampling and DNA Extraction

Colonies of *V. germanica* were sampled in South Africa between 2014 and 2024, from which 48 individual workers were selected to cover the entire geographical range of the species in the country (Table S1, Figure 1). Each worker was sourced from a distinct colony, ensuring that the sample included one individual per colony. After collection from the field, specimens were stored individually in 99% ethanol at −20°C until DNA extraction. Biological tissue was excised from small sections of the thorax of each specimen for independent DNA extraction, then frozen in liquid nitrogen and ground into fine particles using a sterile pestle. DNA extraction was performed using the Qiagen DNeasy Blood & Tissue Kit (Qiagen) according to the manufacturer's instructions, with an additional washing step with 70% ethanol. The final DNA extracts (two per individual) were shipped to CD‐Genomics (www.cd‐genomics.com) in isothermal package with ice packs for 2b‐RAD sequencing.

### 2.2 Mitochondrial Haplotyping

Mitochondrial haplotypes were previously determined for 40 of the 48 colonies from which individual wasps were selected for 2b‐RAD sequencing in this study. As mitochondrial haplotype identification can be achieved by sequencing a short fragment of Cytochrome c oxidase subunit 1 (COI), the remaining eight samples were sequenced for this marker as previously described (van Asch et al. 2022). Each individual's haplotype was determined by sequence alignment with two known COI sequences representing the mitochondrial haplotypes reported in South Africa by van Asch et al. (2022): haplotype H1 (OM735804) and haplotype H2 (OM735805) (Table S1). Multiple sequence alignments were performed using the MAFFT algorithm (Katoh et al. 2009) in Geneious Prime v2023.2.1 (www.geneious.com).

### 2.3 SNP Detection and Nuclear Genotyping

#### 2.3.1 Library Preparation for 2b‐RAD Sequencing

The quality and concentration of each DNA extract and the selection of the best of the two samples from each individual wasp for 2b‐RAD sequencing were performed by CD‐Genomics. Libraries were prepared in accordance with the protocol established by Wang et al. (2012). DNA extracts with concentrations ranging from 100 to 250 ng/μL were digested with the restriction enzyme BsaXI (New England Biolabs, Ipswich, USA) at 37°C for 3 h. The digested fragments were then ligated by mixing with specific adapters (0.2 μM), ATP solution (0.5 mM), T4 DNA ligase enzyme, T4 ligation buffer (all supplied by New England Biolabs) and nuclease‐free water at 16°C for 1 h. Subsequently, PCR amplification was performed to introduce barcodes using specific primers. The amplified products were then purified on 8% polyacrylamide gel. Finally, all individual libraries were combined to form a final composite library, with each component added in equal proportions to ensure uniform representation for subsequent sequencing on an Illumina HiSeq 2500 platform.

#### 2.3.2 Quality Control and Processing of 2b‐RAD Sequencing Data

The sequencing of the 2b‐RAD libraries for the 48 individuals generated approximately 273 million raw reads, averaging 5.45 million reads per individual. Quality control of the sequences was performed by CD‐Genomics using the FastQC tool (Simon 2011; Table S2). The data received in FASTQ format were cleaned through a series of processing steps, including sequence trimming to remove adapters and sample demultiplexing, according to the methods described by Chen et al. (2018).

The clean reads were uploaded and analysed using the Galaxy France platform (usegalaxy.fr). The reference genome alignment method was selected due to its superior performance compared with *de novo* approaches (Rochette et al. 2019). The *V. germanica* (from the UK) genome, iyVesGerm1.1 genome assembly (Crowley et al. 2023), was used as a reference for all subsequent analyses involving SNP positions in the assembly. Alignment of reads to the reference genome was performed using *Bowtie2* (Langmead and Salzberg 2012), and the resulting BAM files were visualised and checked with IGV to ensure alignment quality. The mapped reads were then sorted according to their genomic positions using *Samtools sort* (H. Li et al. 2009). A catalogue of loci was constructed from the sorted BAM files using the *reference map* tool in STACKS2 (Stacks v2.55 + galaxy4). SNPs and genotypes were called using *gstacks* to generate a VCF file. Finally, the *population* tool was used to convert the data into format compatible with STRUCTURE v2.3.4 (Pritchard et al. 2000) and format compatible with Genepop (Rousset 2017). Data filtering in the population tool was configured to balance maximising the number of loci with maintaining reliable SNP coverage. Poorly represented loci and potential alignment errors were excluded to minimise biases in the analysis. The minimum percentage of individuals required in a population to process a locus (‐‐min‐samples‐per‐pop) was set to 0.7, and a locus needed to be present in at least one population (‐‐min‐populations) to be included in the analysis. The minimum percentage of individuals across populations required to process a locus (‐‐min‐samples‐overall) was set to 0.3. The minimum minor allele frequency required to process a nucleotide site at a locus (‐‐min‐maf) was set to 0.05, and the minimum allele count (‐‐min‐mac) was set to 1. The maximum observed heterozygosity required to process a nucleotide site at a locus (‐‐max‐obs‐het) was set to 0.5.

### 2.4 Genetic Structure and Diversity

After quality filtering and variant calling using the *V. germanica* reference genome, STACKS produced a GENPOP file with 2719 RAD loci (one‐locus grain) and a variant‐site dataset with 4910 individual SNP sites. We used the reduced, one‐SNP‐per‐locus GENPOP set for clustering to meet the independence assumption of STRUCTURE, while diversity statistics (*π*, rare alleles) and pairwise genetic distances for subsequent genetic differentiation analyses were computed on the larger 4910‐site dataset to maximise site‐level resolution and lower sampling variance. The SNP final dataset contained 47 individuals, as individual A08 was removed due to high amount of missing data across loci.

The STRUCTURE analysis was conducted utilising the 2719 RAD loci with the following parameters: admixture model, correlated allele frequencies, burn‐in period of 30,000 iterations and 100,000 MCMC iterations. Ten replicates were performed for each proposed cluster (K = 1–8). The number of clusters was determined based on the value of K using the Delta K method (Evanno et al. 2005), and the mean log‐likelihood [LnP(K)] ± standard deviation from 10 replicates per K, calculated with StructureSelector (Li and Liu 2018). All iterations were aligned using the Greedy algorithm implemented in CLUMPAK (Kopelman et al. 2015). The final bar plots were generated with Structure Plot V2.0 (Ramasamy et al. 2014).

A discriminant analysis of principal components (DAPC) was performed in R (R Core Team 2024) using the adegenet package (Jombart 2008) and the dapc() function. To assess genetic structure, up to 10 clusters were tested with 10 principal components (PCs). Based on the K‐statistics, an elbow at two clusters was observed, leading to the selection of three clusters for further analysis. The final analysis retained 35 PCs, accounting for approximately 80% of the total variance and was conducted with the groups identified in previous steps.

To analyse the genetic diversity of the variable sites within the inferred genetic clusters, the number of private alleles, the percentage of polymorphic loci and nucleotide diversity (*π*) were calculated for variable positions in Stacks2: *populations*. Expected heterozygosity (*He*_*exp*_), observed heterozygosity (*He*_*obs*_), genetic differentiation (*F*_*ST*_) between clusters and the inbreeding coefficient (*F*_*IS*_) for each cluster were estimated using the Weir and Cockerham method (Weir and Cockerham 1984), implemented in R with the hierfstat package (Goudet 2005). Confidence intervals (95%) were calculated using a non‐parametric bootstrap with 999 replicates (function boot.ppfst). To test against the null hypothesis of no genetic structure, a permutation test (999 permutations) was performed by randomly shuffling population labels among individuals.

To explore genetic relationships, unrooted Neighbour‐Net phylogenetic trees were built from genome‐wide nuclear SNPs of 47 individuals (4910 variant sites). The SNP dataset was converted to nucleotide format via the *Stacks2 populations* module, yielding a 4910 bp PHYLIP alignment. Analyses were conducted in SplitsTree App 6.0.0 (Huson and Bryant 2024) with pairwise genetic distances calculated using the P‐distance method (Hamming 1950) and 1000 bootstrap replicates for split support. Ambiguous states were handled via two approaches (the default ‘Ignore’ and ‘Average States’) to compare outcomes and balance precision against potential inaccuracies from retained data.

To check the concordance between mitochondrial (haplotypes) and nuclear (SNP‐based clusters) genome affiliations, a chi‐squared test was applied to a contingency table (Table S1) crossing mitochondrial haplotypes and clusters. The test evaluated the null hypothesis (H_0_) of no association between the two types of affiliation.

### 2.5 Drivers of Genetic Differentiation

The geographical coordinates of the sampled colonies were integrated into a Geographic Information System (GIS). For each sampling point, environmental variables from 30‐year annual averages (Schulze et al. 2007) were derived, including precipitation (rmean), mean annual temperature (average: tmean, minimum: tminave, maximum: tmaxave, and summer maximum temperature: tmaxsum and minimum winter temperature: tminwint) and summer normalised difference moisture index (NDMI for 2016 and 2019) (Tables S3 and S6). NDMI for the hottest months (January–March) in 2016 and 2019 was used to represent a wet spring year with typical high moisture conditions, and a dry spring year with low moisture conditions, respectively. These variables were extracted from high‐resolution raster layers and aligned with the colonies using QGIS Version 3.40.5 (QGIS.org 2025). The geographic distance between colonies (dist_geo_km), as well as their distance from the introduction site in Cape Town (dist_km_CT) and the potential secondary introduction site in Stellenbosch (dist_km_SB), were calculated for each sampling point. The geographic locations of the putative invasion centres in Cape Town and Stellenbosch were selected arbitrarily. For Cape Town, the reference point used was the Kirstenbosch National Botanical Garden (−33.9875, 18.4327), where the species was first recorded. In Stellenbosch, the reference point was set at Coetzenburg (−33.9463, 18.8717), close to the town of Stellenbosch, where a population with a high colony density was detected in 2016—likely the result of several years of local establishment. This area was also chosen because both mitochondrial haplotypes H1 and H2 are present there. Absolute differences (delta_) in environmental variables were calculated for each colony pair (e.g., delta_tminwinter representing the difference in minimum winter temperatures), quantifying spatial variation in conditions potentially influencing dispersal success via active flight, human‐assisted spread, or establishment success.

Pairwise *F*_*ST*_ values, based on allele frequencies, were calculated for each pair of individuals using the Stacks2 *populations* module with the ‐fstat option to assess genetic differentiation among *V. germanica* colonies. To investigate the factors influencing genetic differentiation (*F*_*ST*_), we employed Shape‐Constrained Additive Models (SCAM) implemented in the R package scam (version 1.2–19; Pya and Wood 2015). The analysis focused on the 47 individuals and resulted in 1081 pairwise comparisons. The SCAM used a quasi‐binomial family with a logit link to account for the bounded and over‐dispersed nature of pairwise *F*_*ST*_ values relative to a simple binomial assumption. The full model incorporated three predictor components: genetic SNP cluster types (clus_type: both P1, both P2, or P1P2), spatial variables (geographic distance between samples, dist_geo_km and differences in distance to introduction sites, delta_dist_km_CT and delta_dist_km_SB) and environmental gradients (temperature differences: delta_tmean, delta_tmaxsum, delta_tmaxave, delta_tminwint; precipitation: delta_rmean; and normalised moisture indices: delta_NDMI16, delta_NDMI19). Shape constraints were applied to ensure that the model captured biologically realistic patterns. In particular, genetic differentiation is expected to rise or level off with geographic or environmental distance, but not to decline. Then, monotonic increasing constraints were applied to geographic distance (dist_geo_km) and temperature gradients (delta_tmean, delta_tmaxsum). For rainfall and moisture indices (delta_rmean and delta_NDMI16), positive constraints were applied to allow variation while preventing negative effects, thus reducing the risk of overfitting by avoiding implausible, non‐biological fluctuations in the fitted curves. In contrast to predictors with constrained effects, the difference in distance to Stellenbosch (delta_dist_km_SB) was included as a linear term in the model. In the initial model run, its estimated degree of freedom (edf) was close to 1, reflecting the lack of specific expectations regarding its effect on genetic differentiation. Model selection involved removing predictors with strong pairwise correlations (> 0.75) and assessing concurvity. The edf for each predictor quantified the nonlinearity of its effect, with edf ≈1 indicating a linear effect, edf > 1 a nonlinear effect, and edf ≈0 a trivial effect after penalisation, leading to its removal from the final model. Model performance was evaluated using residual diagnostics and deviance explained.

Isolation by distance was also tested using the mantel.randtest function from the adegenet (Jombart 2008) package (v2.1.5) in R Genetic distances were computed from multilocus genotypes and geographic distances as Euclidean distances from sampling coordinates. The Mantel test was applied to the full dataset and to each identified genetic cluster (10,000 permutations). Mantel test results were visualised with scatter plots of geographic versus genetic distances and density plots generated using the kde2d function from the MASS package (Ripley et al. 2013).

To assess the unique and shared effects of spatial, environmental and genetic components on genetic differentiation (*F*_*ST*_), we applied hierarchical variation partitioning using deviance explained from SCAM. Reduced models were obtained by removing one component (spatial, environmental, or genetic) from the full model, while simple models included a single component. The unique contribution of each component was calculated as the difference in deviance explained between the full model and the corresponding reduced model. The shared contribution of two components was calculated as the sum of the deviance explained by their simple models minus the deviance explained by the reduced model of the third component. To assess whether the contribution of each predictor component to *F*_*ST*_ was greater than expected by chance, we performed 1000 permutation tests.

To compare *F*_*ST*_ values between identified SNP clusters, we used a Kruskal–Wallis test (Kruskal and Wallis 1952). When significant, post hoc pairwise comparisons were performed with Dunn's test (Dunn 1964) applying a Bonferroni correction for multiple testing using the R package dunn.test (Dinno and Dinno 2017). Climatic differences between genetic clusters were assessed using permutation tests. For each SCAM environmental variable of interest (rmean, NDMI16, tmean and tmaxsum), we calculated the observed mean difference between clusters, followed by 10,000 permutations in which cluster labels were shuffled while climate values remained fixed. Two‐sided *p*‐values were derived as the proportion of permuted differences equal to or more extreme than the observed.

## 3 Results

### 3.1 SNP Detection and Genetic Structure Analyses

**FIGURE 2:**
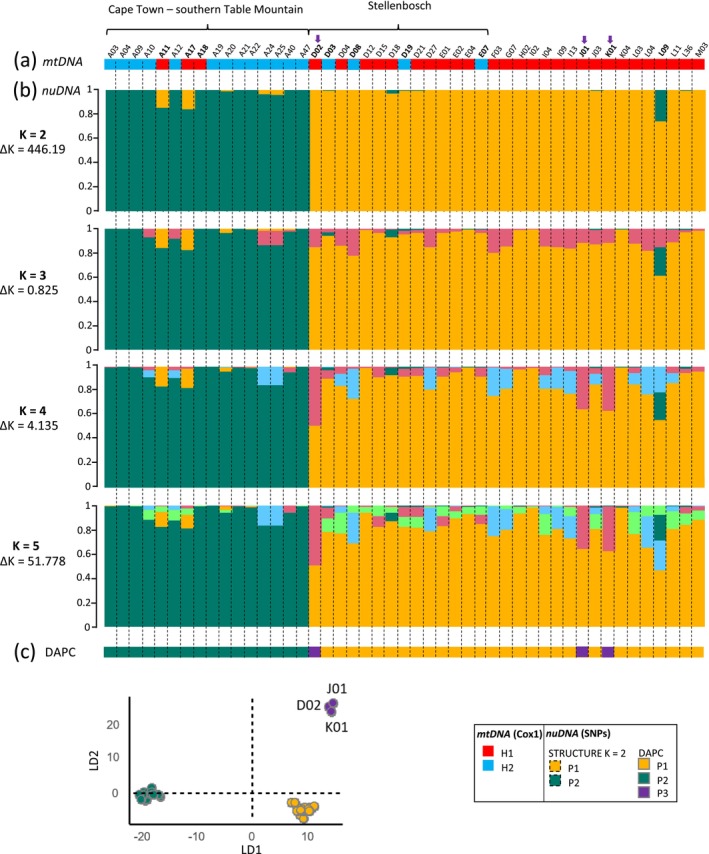
Genetic structure of 47 individual wasps of *Vespula germanica* collected in South Africa (*n* = 47). (a) Mitochondrial haplotype associated with each individual. (b) Population structure analysis of nuclear SNPs using STRUCTURE, showing the number of clusters for K = 2–5. Each bar represents one individual, with the probability of belonging to each cluster displayed on the y‐axis. (c) Results of the DAPC (discriminant analysis of principal components) of the nuclear SNP data. Individuals written in bold are mentioned in the text. Individuals marked with an arrow belong to sub‐group P3.

STRUCTURE analysis was performed on 2719 RAD loci from 47 individuals, excluding A08 due to excessive missing data. The Evanno method (ΔK) supported K = 2 (ΔK = 446.19), indicating two main genetic clusters. Nevertheless, log‐likelihood values continued to increase up to K = 5 (mean LnP(K) = −98873.43), pointing to possible substructure (Figure 2b, Figure S1). At K = 4 and K = 5, three individuals (D02, J01, K01) showed apparent mixed ancestry with assignment to both the main P1 component (yellow) and a secondary component. DAPC (Figure 2c) clearly grouped these three samples into a small, cohesive cluster (hereafter P3). This contrast likely reflects methodological differences: DAPC, which does not assume Hardy–Weinberg equilibrium and is well suited to detecting small distinct clusters, identifies P3 as a locally differentiated group, whereas STRUCTURE often struggles to detect very small clusters and can portray them as admixed when only a few samples are available. Thus, we recognised three groups overall: P1 (*n* = 28), P2 (*n* = 16) and P3 (*n* = 3). The three outlier individuals in P3 were sampled in different sites: D02 in Stellenbosch, J01 in Worcester (east) and K01 in Kleinmond (south). These locations are 45–77 km apart and separated by mountainous relief (Figure 1).

**FIGURE 3:**
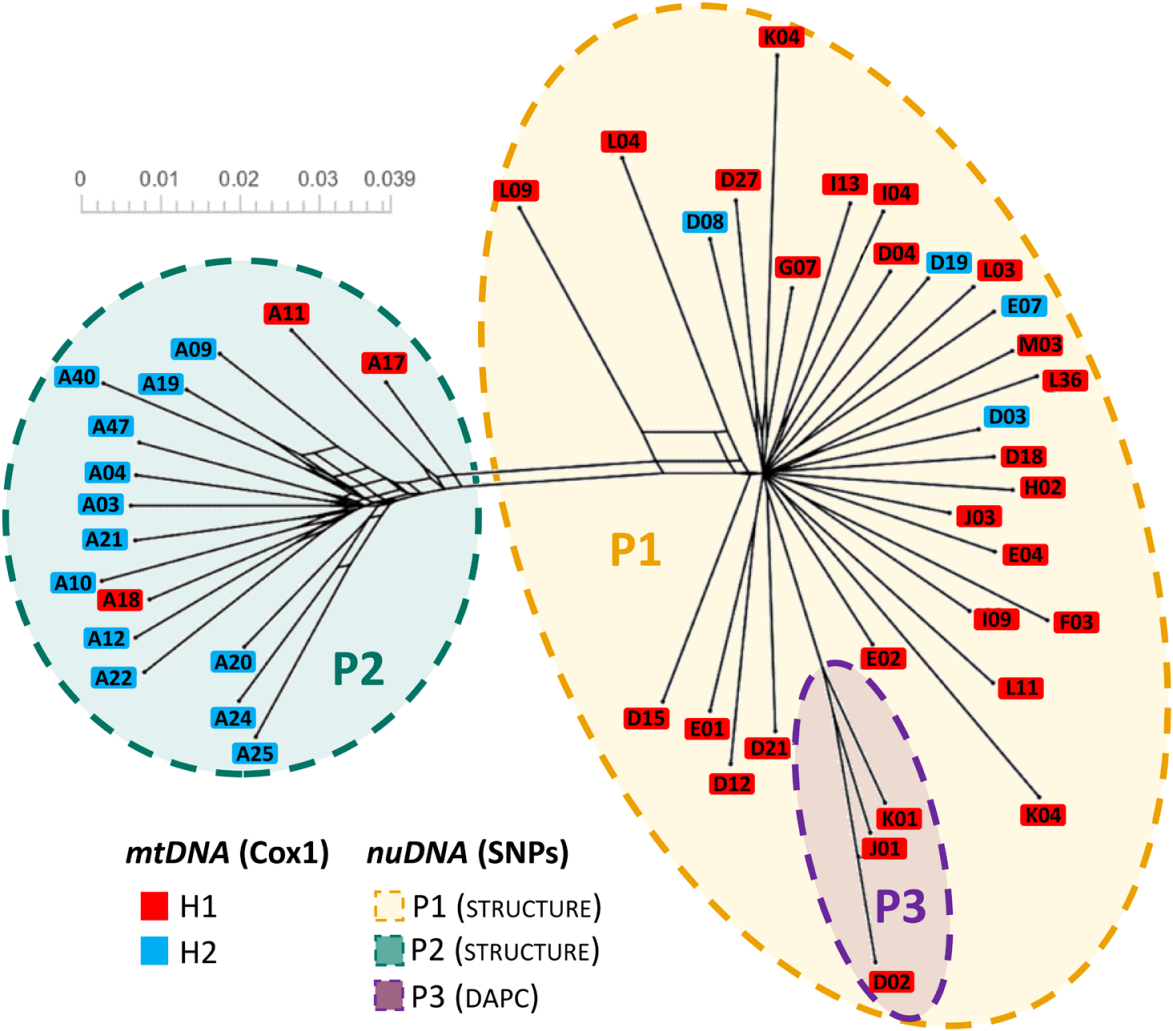
Neighbour‐net phylogenetic network of *Vespula germanica* collected in South Africa, based on genome‐wide SNP data for 47 individuals (4910 sites). The network was generated on SplitsTree using the ‘Handle Ambiguous States = Ignore’ option and achieved a Fit of 99.8% (1000 bootstraps). The 129 splits, visualised as parallel or intersecting lines, show reticulations and conflicting phylogenetic signals. The colours represent mitochondrial and nuclear genetic affiliations. The scale bar represents genetic distance.

The Neighbour‐Net tree reveals a clear phylogenetic structure among the individuals and supports the genetic clusters identified by STRUCTURE and DAPC analyses. Within the larger P1 cluster, the distinct sub‐cluster P3 emerges, formed by individuals K01, J01 and D02, corresponding to the third cluster P3 identified by DAPC (Figure 3). These individuals also share a common genetic ancestor with P1, as indicated by the STRUCTURE analysis. The tree exhibits multiple reticulations, particularly near the central nodes of P2 (Figure 3).

Given the small size of cluster P3 and its strong genetic affinity with P1, subsequent association and diversity analyses were performed either by merging P3 with P1 (hereafter P1 + 3) or by treating P3 as a separate group.

The chi‐squared test revealed a significant association between mitochondrial haplotypes and genetic clusters (*χ* ^2^ = 18.49, *p* ≤ 0.001) (Figure 2b and Figure S1). Individuals carrying mitochondrial haplotype H1 are assigned to genetic cluster P1 + 3 (*N*_P1 + 3_ = 31) and those carrying haplotype H2 are assigned to genetic cluster P2 (*N*_P2_ = 16), with the exception of four individuals (A11, A17, A18) from the Cape Town area that carry haplotype H1 but were assigned to P2, and four individuals (D03, D08, D19 and E7) from the Stellenbosch area that carry haplotype H2 but were assigned to P1 (Table S1, Figures 1 and 2). At K = 2, individuals A11 and A17 from cluster P2 showed proportions of 0.14 and 0.16 assigned to cluster P1 + 3, respectively, while individual L09 from cluster P1 + 3 had a proportion of 0.26 assigned to cluster P2 (Figure 2b).

Analyses of genetic clusters show higher genetic diversity in P1 + 3 compared with P2 (Table 1). P1 + 3 exhibits more private alleles (650–1015 vs. 331), higher nucleotide diversity (*π*) and greater expected and observed heterozygosity (*He*_*exp*_, *He*_*obs*_), but a higher *F*_*IS*_ (0.130–0.137 vs. 0.078), indicating slight inbreeding. Overall (*n* = 47), the population shows substantial genetic diversity (*π* = 0.295, *He*_*obs*_ = 0.231) (Table 1). The genetic differentiation between P1 + 3 and P2 shows significant divergence (*F*_*ST*_ ≈ 0.15; 95% CI = [0.14–0.16]), falling within the ‘moderate’ range according to Wright's qualitative classification (Wright 1965).

**TABLE 1:** Genetic diversity and inbreeding statistics for nuclear‐DNA SNP genetic clusters P1, P2 and combined P1 + 3 (including P1 and sub‐cluster P3) inferred by STRUCTURE and DAPC.

Clusters	*n*	Private alleles	% polymorphic loci	*π*	*He*_*exp*_	*He*_*obs*_	*F*_*IS*_ [95% CI]
P1 + 3	29	1015	80.48	0.282 ± 0.002	0.273	0.232	0.137 [0.126–0.148]
P1	26	650	80.49	0.282 ± 0.002	0.272	0.233	0.130 [0.119–0.141]
P2	18	331	82.09	0.257 ± 0.002	0.253	0.231	0.078 [0.065–0.092]
Overall (P1 + 3 + P2)	47	0	79.70	0.295 ± 0.002	0.263	0.231	0.110 [0.102–0.118]

Abbreviations: % polymorphic loci, Percentage of loci with multiple alleles; *F*_*IS*_, Inbreeding coefficient with 95% confidence interval; *He*_*exp*_, Expected heterozygosity under Hardy–Weinberg equilibrium; *He*_*obs*_, Observed heterozygosity; *n*, number of individuals; Private alleles, Alleles unique to each cluster; *π*, Nucleotide diversity (±SE).

### 3.2 Effects of Geographic Distance and Environmental Conditions

**FIGURE 4:**
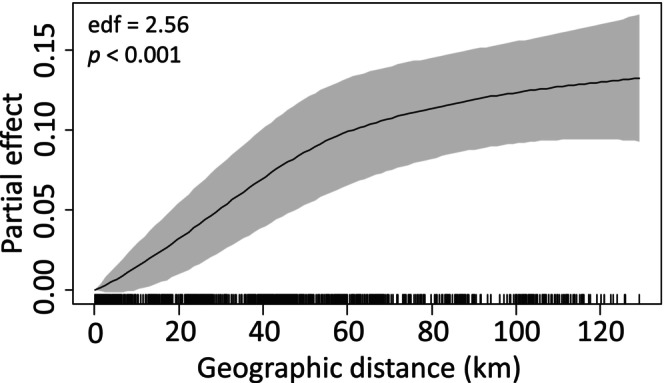
Nonlinear partial effect of geographic distance on genetic differentiation (*F*_*ST*_) in *Vespula germanica* in South Africa. Smoothed curve (smooth term) from the SCAM model (quasi‐binomial, logit link) for the geographic distance (variable dist_geo_km) in km between pairs of individuals (*n* = 1081 pairs, 47 individuals). Steeper slopes indicate greater influence on genetic differentiation. The lines at the bottom of the x‐axis indicate the observed values of geographic distance for the pairs of individuals.

To identify the factors that best explain pairwise genetic differentiation (*F*_*ST*_) between colonies, we fitted a shape‐constrained additive model (SCAM) that regresses *F*_*ST*_ on three predictor components: genetic cluster identity (P1 + 3 or P2), spatial variables (geographic distances between pairs of individuals and distances to putative introduction sites) and environmental dissimilarities (temperature, precipitation, moisture indices). After removing all environmental smooth terms penalised to edf = 0 (indicating negligible effects), the final model explained 56.3% of the deviance (adjusted *R* ^2^ = 0.560, *n* = 1081 pairwise comparisons) (Table S4). The smooth term for geographic distance was highly significant and nonlinear (edf = 2.56, *F* = 26.61, *p* < 0.001), consistent with isolation by distance (IBD): the partial effect of geographical distance increased sharply up to ~50 km before flattening (Figure 4). The linear effect of distance to Stellenbosch was also significant (estimate = −0.0007, *t* = −3.322, *p* < 0.001), indicating that genetic differentiation was lower between pairs differing strongly in their distance to the hypothesised introduction point in Stellenbosch. SNP Cluster type had strong effects: Compared with pairs within P1 + 3 (reference), pairs involving P1 + 3 and P2 showed much greater genetic differentiation (estimate = 0.172, *t* = 22.04, *p* < 0.001), whereas P2–P2 pairs showed reduced differentiation (estimate = −0.078, *t* = −6.20, *p* < 0.001) (Table S4). Model diagnostics confirmed full convergence after 10 iterations (scale estimate = 0.003). Without accounting for the nonlinear IBD effect, no significant correlation between genetic and geographic distances was detected using Mantel tests with 10,000 permutations: P1 + 3 (*r* = −0.063, *p* = 0.668), P2 (*r* ≈ 0.000, *p* = 0.252), or the full dataset (*r* = 0.186, *p* = 0.059; marginally non‐significant) (Figure S3).

Hierarchical partitioning of *F*_*ST*_ variation revealed that genetic clustering had the largest unique and significant contribution (28.9%, *p* = 0.006), followed by significant but small spatial effects (3.0%, *p* = 0.001), while environmental gradients contributed negligibly (~0%, *p* = 0.989) (Table S5). Substantial shared variance highlighted strong collinearity: environmental effects overlapped largely with genetic clustering (45.3%) and spatial factors (39.0%) (Table S5). In short, the SCAM shows that genetic clustering is the dominant predictor of *F*_*ST*_, geographic distance adds modest independent IBD signal at short range and environmental differences mostly co‐vary with cluster identity.

**FIGURE 5:**
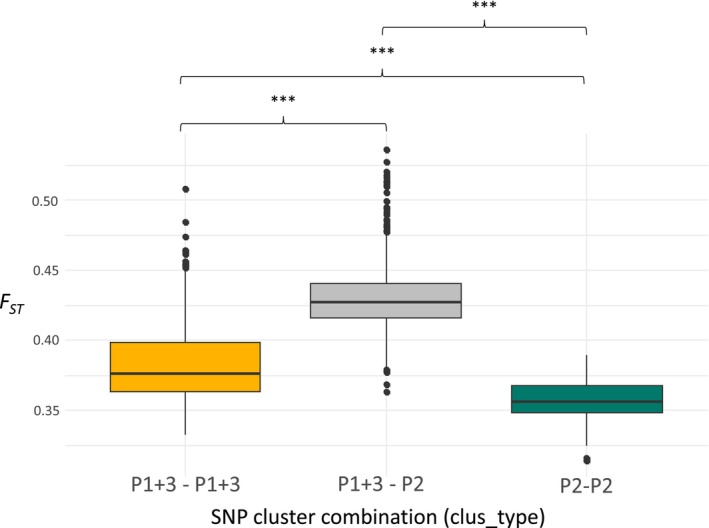
Genetic differentiation (calculated as *F*_*ST*_ from genome‐wide SNP data) among *Vespula germanica* sampled in South Africa (*n* = 47) according to pairs of individuals from the two main clusters based on nuclear SNPs (P1 + 3 and P2). Boxplots show the distribution of *F*_*ST*_ values between pairs of individuals (*n* = 1081). Asterisks above brackets indicate significance levels from Kruskal–Wallis tests (***p < 0.001).

The Kruskal‐Wallis test followed by Dunn's post hoc showed that P1 + 3‐P2 pairs had significantly higher *F*_*ST*_ values than both P1 + 3–P1 + 3 (*Z* = −19.96, *p* < 0.001) and P2‐P2 (*Z* = 7.04, *p* < 0.001) pairs. A significant difference was also detected between P1 + 3–P1 + 3 and P2‐P2 pairs (*Z* = 19.75, *p* < 0.001) (Figure 5).

**FIGURE 6:**
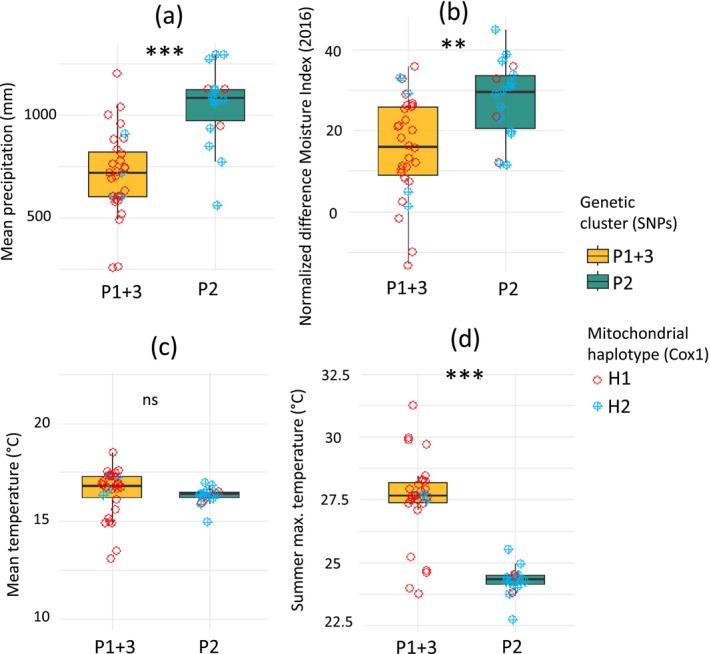
Comparison of environmental variables between genetic clusters of *Vespula germanica* sampled in South Africa. (a) Mean precipitation (mm), (b) Normalised Difference Moisture Index, (c) mean temperatures (°C) and (d) maximum summer temperatures (°C) measured at the sampling sites of the 47 individuals. Asterisks indicate significance levels based on 10,000 permutations (ns: Non‐significant, **p < 0.01; ***p < 0.001).

Precipitation (rmean) and maximum summer temperature (tmaxsum) showed the strongest differentiation between the geographic ranges of the clusters (*p* < 0.001). The Normalised Difference Moisture Index (NDMI16) also differed significantly, though less extreme (*p* = 0.0017), while mean temperature showed no significant difference (*p* = 0.504) (Figure 6).

## 4 Discussion

The European wasp *V. germanica*, introduced to South Africa in the 1970s, has exhibited a remarkably slower spread compared with other invaded regions. Our SNP analyses (2b‐RAD, 47 colonies) identified two to three genetic clusters concordant with mitochondrial haplotype and associated with environmental variables, offering insights into how ecology, dispersal, traits and introduction history influence invasion dynamics.

### 4.1 Dual Introductions and Limited Effective Gene Flow Between Lineages

SNP‐based population structure analyses identified two main genetic clusters, P1 and P2, with a finer substructure (P3) nested within P1. P3 represents only three individuals (~6%) not grouped geographically. The distinct genetic signature of P3 is best explained by a local founder event followed by genetic drift, producing a small, differentiated subset of the P1 background without requiring a separate introduction. Human‐mediated movement likely dispersed these newly differentiated P3 individuals to multiple sites. Evolutionary invasion models and empirical studies show that small founder bottlenecks can rapidly generate stochastic genetic differences (Nei et al. 1975), a phenomenon known to influence the spread and establishment of invasive populations in social insects (Hagan et al. 2024).

The broader nuclear genetic structure (P1 + 3 vs. P2) shows a clear east–west split: P1 + 3 individuals occur broadly across the geographical range, whereas P2 is restricted to the Kirstenbosch–Table Mountain area. Both genetic clusters display heterozygosity deficits consistent with invasion bottlenecks and drift (Sakai et al. 2001; Sherpa and Després 2021). A Wahlund effect (i.e., undetected substructure within sampled groups) could also contribute to reduced heterozygosity, but the clear subdivisions of genetic groups (P1 +3 vs. P2) support that demographic processes are the primary drivers of the observed heterozygosity deficits. The deficit is more pronounced in P1 + 3, potentially due to heterogeneous landscape barriers creating substructure, whereas P2's more homogeneous environment may reduce inbreeding. This genetic structure differs slightly from the mitochondrial pattern: although haplotypes H1 and H2 co‐occur in both Cape Town and Stellenbosch, genetic clusters based on nuclear SNPs show strict spatial segregation.

The dominant mito‐nuclear associations (H1 with P1 + 3 and H2 with P2) support the dual‐introduction hypothesis proposed by van Asch et al. (2022). This pattern distinguishes between two neutral demographic scenarios: two separate introductions from distinct sources, which create linked mito‐nuclear lineages, versus a single introduction followed by population subdivision and drift. While drift can generate correlated allele frequency shifts across loci (Barrett and Schluter 2008; Estoup and Guillemaud 2010), the strong mito‐nuclear concordance is more parsimoniously explained by dual introductions. Indeed, differences in effective population size, inheritance and recombination rates between genomes make it unlikely that drift alone would produce and maintain such clear mito‐nuclear concordance after a single founding event (Ballard and Whitlock 2004). We therefore conclude that the east–west genetic split most plausibly originated from two independent introductions.

The maintenance of this strong genetic structure, despite some dispersal, indicates the presence of contemporary barriers to gene flow. SCAM analyses show a clear IBD pattern: genetic differentiation increases sharply with geographic distances and then levels off beyond ~40–50 km, a pattern typical of social insects (Ross 2001; Ross and Shoemaker 1997). Male behaviour, which consists of aggregating near nests and engaging in lek‐ or swarm‐like mating (Martínez et al. 2018; Masciocchi et al. 2020), is compatible with limited long‐range nuclear homogenisation. Nevertheless, occasional mito‐nuclear discordance (e.g., H1 with a P2 nuclear background and vice versa) and three individuals (A11, A17, L09) showing > 10% ancestry in the alternate cluster show that long‐distance movement and episodic admixture occur, likely involving human‐mediated transport of gynes (Masciocchi and Corley 2013). Because gynes mate before hibernation, transported mated gynes retain their maternal haplotype while descendants can gradually acquire the local nuclear background through subsequent mating and backcrossing, producing mito‐nuclear discordance (e.g., H2 haplotypes with P1 + 3 at Stellenbosch, or and H1 haplotypes with P2 at Cape Town–Table Mountain). While admixture is known to facilitate invasion by enhancing genetic diversity (Diedericks et al. 2018; Fournier and Aron 2021; Prentis et al. 2008), its low frequency here (6% of individuals) is consistent with the slow invasion dynamics observed in South Africa (Sherpa and Després 2021).

However, the limited gene flow is not solely due to a lack of dispersal. Indeed, the geographic ranges of H1‐P1 + 3 and H2‐P2 overlap, suggesting that reproductive barriers or selection against hybrids may hamper successful admixture. Assortative mating, particularly between H1 gynes and P1 + 3 males, may be an alternative explanation for the observed nuclear mito‐nuclear genetic structure: assortative mating has been reported in social insects, e.g., mate choice based on cuticular hydrocarbons (CHCs) in *Formica* ants (Blacher et al. 2022). In *V. germanica*, as in other social wasps where males aggregate near colonies (Martínez et al. 2018; Masciocchi et al. 2020), such mating preferences could reinforce kin structure and, after a bottleneck, paradoxically maintain low genetic diversity and limit spread. Nonetheless, assortative mating remains only one of several possible explanations for the observed genetic structuring. Tests such as behavioural assays, paternity analyses, or trait‐genotype correlations (e.g., CHCs or facial patterns linked to haplotypes) are needed to evaluate this hypothesis.

### 4.2 Ecological Pre‐Adaptation Maintains Genetic Structure and Constrains the Spread

While the SCAM confirms significant IBD, the unique contribution of geographic variable is modest (≈3%), and the complementary Mantel tests found no clear IBD. Nevertheless, the nuclear genetic structure explains a substantial unique portion of genetic differentiation, strongly overlapping with the environmental component, indicating a tight association between genetic clusters and habitat. Specifically, differences in summer maximal temperature and moisture, rather than annual mean temperature, best distinguish the two main genetic clusters: P1 + 3 occurs in hotter, drier sites while P2 occupies cooler, wetter sites. Previous species distribution models (SDMs) identified a general preference of the species for cooler, moister conditions in the region (Veldtman et al. 2021) and noted that irrigation systems could expand the wasp's potential range (De Villiers et al. 2017). Our genomic results refine this understanding by demonstrating that these environmental constraints correspond to genetically distinct clusters, each associated with a specific habitat.

The strong association between nuclear genetic clusters and distinct climatic niches supports an overall pattern of isolation by environment (IBE), a process in which genetic differentiation increases with environmental differences independently of geographic distance (Wang and Bradburd 2014). This pattern, however, is shaped at the broad scale among clusters and does not necessarily imply strong IBE processes acting within each cluster. For instance, the low genetic differentiation within cluster P2 likely reflects this adaptation to cool, humid environments, possibly linked to metabolic efficiency, but this homogeneity may also stem from their restricted urban distribution around Cape Town, which facilitates both natural dispersal and human‐mediated transport. Conversely, the broader niche occupancy of P1 + 3 may also be aided by its higher gene‐frequency diversity, which can enhance adaptive responses (Caballero and García‐Dorado 2013).

For IBE to maintain such genome‐wide structure, mechanisms must restrict gene flow between contrasting habitats. Although several *Vespula* species show substantial phenotypic plasticity (e.g., *Vespula vulgaris*, Badejo et al. 2020; *Vespula maculifrons* , Orr et al. 2024), some traits, particularly in queens, also show measurable heritability (Orr et al. 2024), which provides the genetic substrate for local specialisation. Thus, pre‐existing ecological and genetic differences between the introduced populations may have been reinforced during invasion by selection on fitness‐related traits (Bossdorf et al. 2005; Hufbauer et al. 2012; Mack 2003; Olivieri 2009), as seen in other wasps (Wilson et al. 2009). This scenario is consistent with the restricted gene flow observed in South Africa, suggests that immigrants or hybrids suffer low fitness in non‐native habitats, a signature of selection against migrants or reduced hybrid viability (Lu and Bernatchez 1999; Räsänen and Hendry 2008). Assortative mating, as discussed earlier, could further strengthen this reproductive barrier (Knight and Turner 2004). Future experimental work or genome‐wide association study on temperature and humidity tolerances will be required to validate these hypotheses.

The concept of isolation reflecting ecological pre‐adaptation aligns with niche conservatism. Although debated, only limited niche expansion between native and introduced ranges has been reported in invasive species, especially for terrestrial ectotherms such as insects (Liu et al. 2020; Aravind et al. 2022). Identifying source populations is thus critical. Comparative mitogenome analyses (van Asch et al. 2022) suggest that H1 (linked to P1 + 3) originated from a source preadapted to Western Cape conditions, while H2 (linked to P2) came from a less compatible source. This hypothesis is supported by Eloff et al. (2020) where haplotype groups were linked to different native regions (e.g., one group linked to French/Argentinian populations and another to Swiss/Austrian/NZ populations), though sequencing artefacts and sampling issues prevented exact matches. Our study provides the first genome‐wide analysis of *V. germanica*. However, both mitochondrial and nuclear data for this species are sparse, which limits the ability to precisely infer the source regions. Broader sampling across the native range, combined with higher‐resolution genomic data, will be required.

Finally, although the SCAM explained 56.3% of the deviance, some variance remained unexplained. This could reflect fine‐scale ecological and anthropogenic factors not captured by our predictors: hydrological preferences (*V. germanica* preferentially nests near watercourses; Veldtman et al. 2021), urban heat‐island effects and resource availability that favour suburban and urban populations (Sorvari 2018; Warren and Promowicz 2025), and differential expansion from putative introduction cores shaped by mountain barriers (Goodisman, Matthews, and Crozier 2001). The observed genetic patterns are influenced not only by broad climate gradients but also by a fine‐grained mosaic of favourable habitats, reflecting the complex ecological and anthropogenic landscape of the Western Cape and placing the South African invasion of *V. germanica* in a distinct category compared with the rapid and homogeneous expansions observed elsewhere.

### 4.3 *Vespula* Invasion Trajectories: Slow, Structured Expansion in South Africa

Within the genus *Vespula*, genetic outcomes of invasion vary considerably. While in native ranges, effective queen dispersal (often facilitated by human transport) commonly results in large‐scale panmixia, as seen in *V. vulgaris* in the UK (Cunningham‐Eurich et al. 2023), some introduced populations, like *V. germanica* and *V. vulgaris* in New Zealand, undergo rapid, homogeneous expansion following a severe genetic bottleneck (Lester et al. 2014; Schmack et al. 2019). Most introduced populations follow divergent trajectories, influenced by multiple introductions and landscape barriers, and develop strong genetic structure differentiated by geography (e.g., *V. pensylvanica* in Hawaii; Chau et al. 2015; Hanna et al. 2014) or by distance (e.g., *V. germanica* in Australia; Goodisman, Evans, et al. 2001). Such structure can be maintained by limited natural gyne dispersal with short flight distances, as in Argentina (Masciocchi and Corley 2013) combined with rare long‐distance jumps, or, as our data indicate, by environmental filtering and adaptation to specific habitats. Moreover, invasion success in *Vespula* is frequently tied to human‐mediated long‐distance dispersal and social plasticity, such as the weak innate nestmate discrimination that facilitates a shift to perennial polygyny in mild climates (Hanna et al. 2014; Loope et al. 2018), although perennial polygyny has not been observed in the South African invasion.

**FIGURE 7:**
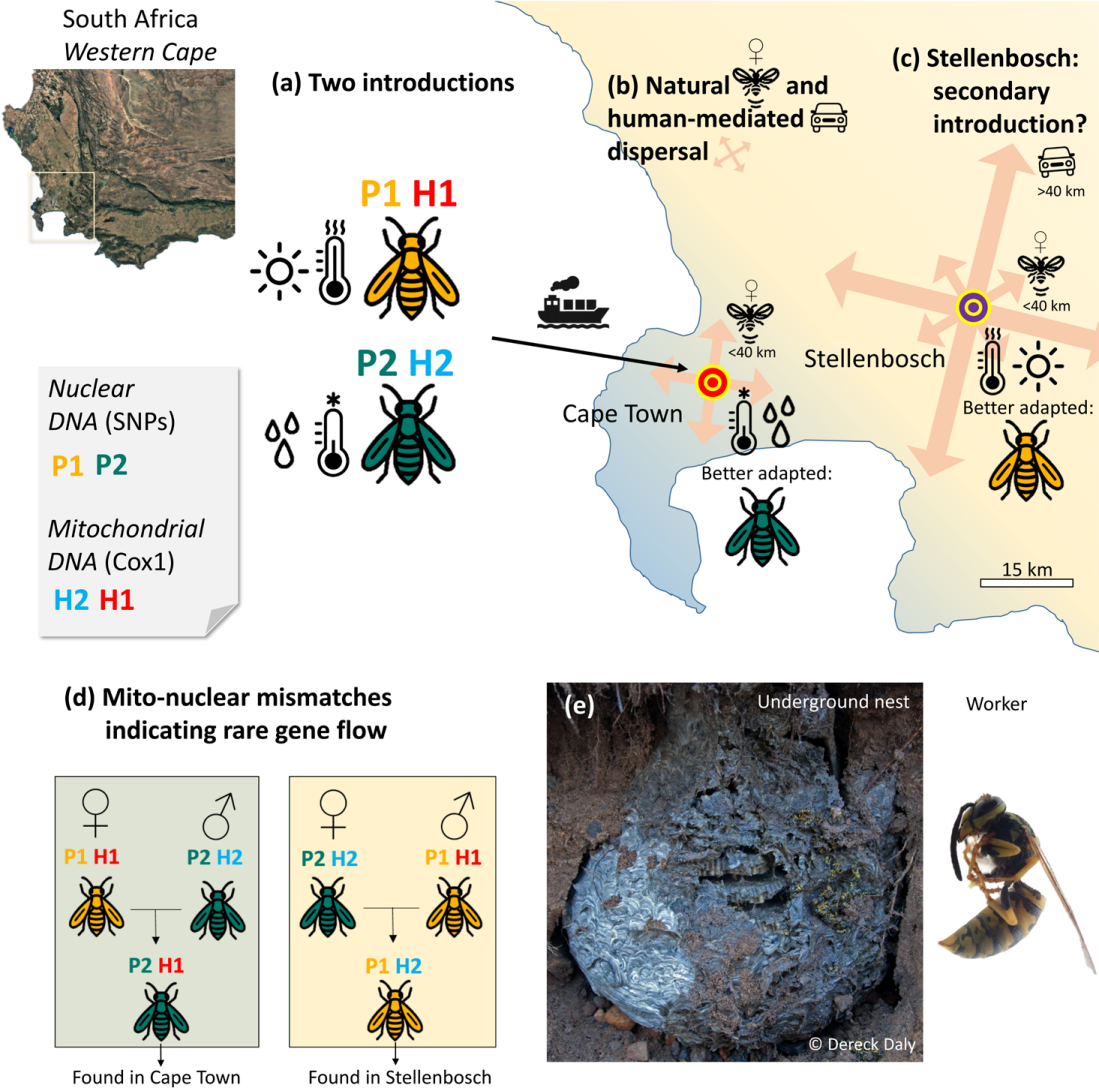
Proposed invasion scenario for *Vespula germanica* in the Western Cape, South Africa. (a) Two genetically distinct populations, each preadapted to specific climatic conditions, were independently introduced into the Western Cape province in South Africa. (b) Their contrasting distributions reflect both limited natural and episodic human‐mediated dispersal. P1 + 3 expanded towards warmer inland regions, whereas P2 remains confined to cooler, wetter microclimates. (c) Stellenbosch may represent a secondary introduction point facilitating further expansion of P1. (d) Occasional gene flow events generating mismatched nuclear‐mitochondrial genotypes (e.g., P2 nuclear genome with H1 mitochondria) were detected. (e) Example of a typical underground nest and a *V. germanica* worker from the study area.

Our results position the South African invasion of *V. germanica* firmly within the category of slow, structured expansion, where limited gene flow and ecological divergence have shaped its unique dynamics. Specifically, the dual‐introduction history, coupled with the strong isolation‐by‐environment signal linking each genetic cluster to distinct climatic niches, provides the foundation for a coherent invasion scenario. We therefore propose that two genetically distinct populations colonised different environmental niches and produced genetic clusters now segregated by the climatic characteristics of the environment (Figure 7a). Subsequent spread has been characterised by short‐range natural movements of gynes and males, generating local genetic viscosity, while rare human‐mediated jumps have led to episodic, low‐level admixture (Figure 7b,d). The broader distribution of the heat‐tolerant cluster (P1 + 3) likely results from a combination of natural gyne flights and human‐assisted transport along corridors, whereas the confinement of the cool‐adapted cluster (P2) to the Cape Town area reflects its narrower niche. Quantifying the relative contributions of each type of dispersal is difficult, as IBD and environmental effects overlap in SCAM; direct movement data would clarify this. The town of Stellenbosch, with strong transport links and a favourable microclimate, may represent a secondary introduction or expansion point (Figure 7c). The sporadic presence of mitochondrial H2 there, linked only to P1 + 3 nuclear backgrounds, likely reflects incidental introductions without establishment, perhaps indicating limited admixture during regional expansion in the 2000s (Veldtman et al. 2021). The timing and sequence of introductions remain unresolved, but broader sampling could allow tests with Approximate Bayesian Computation (ABC) (Csilléry et al. 2010; Lombaert et al. 2011).

### 4.4 Towards a Predictive Framework for Social‐Insect Invasions: Lessons From *V. germanica*

Although *V. germanica* is a globally successful invader, its performance varies markedly between regions. Our results illustrate a central tenet of invasion ecology: success is context‐dependent, shaped by the interaction of species traits, recipient‐ecosystem properties and introduction history (Blackburn et al. 2011; Facon et al. 2006), a combination conceptualised as the invasion syndrome (Kueffer et al. 2013; Novoa et al. 2020; Pyšek et al. 2020). For *V. germanica* in South Africa, the resulting invasion syndrome is a slow, structured expansion, explained by the combination of dual introduction, environmental filtering (IBE) and a dispersal system dominated by short‐distance movement and rare successful human‐assisted jumps.

Modern genomics has revealed a broad spectrum of invasion genetic patterns in insects (Tay and Gordon 2019; Webster et al. 2023). Some invasive insects, like the diamondback moth (*Plutella xylostella*), show panmixia at the intercontinental scale due to frequent long‐distance dispersal (Perry et al. 2020), while others, like certain mosquitoes, exhibit structure heavily influenced by human transportation networks (Schmidt et al. 2020). Many invasive species thrive despite bottlenecks (Lombaert et al. 2025), and some wasps achieve rapid global spread via multiple introductions (Dittrich‐Schröder et al. 2018). The South African *V. germanica* invasion represents a distinct point on this spectrum: its genetic structure is environmentally anchored, demonstrating how potent niche filtering can produce a predictable, slow syndrome even in a dispersive species.

The unique interplay of traits in social insects (colonial life, reproductive division of labour and often complex communication) introduces a new layer of complexity to invasion dynamics. While eusociality promotes local resilience and competitive dominance (Bertelsmeier 2021; Chapman and Bourke 2001; Holway et al. 2002; Liu and Stiling 2006), it may also impose constraints that reduce effective population size and genetic diversity. For instance, we propose that assortative mating could help maintain the observed genetic structure in *V. germanica*. In Hymenoptera such as *V. germanica*, low genetic diversity can increase production of sterile diploid males homozygous at the complementary sex determiner (csd) locus, threatening colony viability (Beye et al. 2003). Therefore, to build predictive frameworks for invasion syndromes, future work must integrate social traits and phenotypes. This could be achieved through comparative studies that contrast the social and genomic profiles of invasive social insects. Such integrative work would improve our ability to predict invasion outcomes and design targeted policy and management actions (Novoa et al. 2020).

## Author Contributions

D.G., R.V., C.H., and B.A. conceived and designed the study. R.V. collected the field samples. D.G. performed the pre‐sequencing laboratory genetic work. B.A. and C.H. provided laboratory equipment and technical advice. G.B. contributed data used in part of the analyses. C.H. assisted with statistical analyses. D.G. performed the data analyses, prepared all figures, and wrote and formatted the original draft. All authors reviewed, edited, and approved the final version.

## Funding

This study was funded by the National Research Foundation (NRF SARChI grant 89967, https://www.nrf.ac.za/) and the Stellenbosch University Subcommittee B. Additional support from the National Institute for Theoretical and Computational Sciences (NITheCS, https://nithecs.ac.za/) is also acknowledged.

## Conflicts of Interest

The authors declare no conflicts of interest.

## Supporting information


**Appendix S1:** mec70217‐sup‐0001‐AppendixS1.zip.

## Data Availability

Supplementary tables, figures and R scripts for the *F*_*ST*_ analyses, SNP dataset and intermediate files are provided in Appendix S1. All documents, as well as the VCF file, are deposited on Figshare (https://doi.org/10.6084/m9.figshare.28890326). Sequencing reads are deposited in the NCBI Sequence Read Archive (BioProject ID PRJNA1368704; http://www.ncbi.nlm.nih.gov/bioproject/1368704).
