# Supplementary material for: Genomic Evidence for Dual Introductions, Limited Gene Flow and Niche Preferences in the Invasive Wasp Vespula germanica in South Africa
Source: Mol Ecol. 2025 Dec 22;35(1):e70217. doi: 10.1111/mec.70217 (PMC12745851; doi:10.1111/mec.70217)
Supplement: Supplementary file 1 — Appendix S1: mec70217‐sup‐0001‐AppendixS1.zip. [file MEC-35-e70217-s001.zip › 1_Gergonne_etal_Supplementary_Tables_Figures.docx]

**Supplementary Tables and Figures**

**Table S1.** List of individual specimens of *Vespula germanica* collected across the geographic range of the species in South Africa. Sampling information, mitochondrial haplotype, and genetic cluster based on 2b-RAD sequencing data are presented. Ref 1: van Asch et al. 2022; ref 2: Eloff et al. 2020

| **Sample code** | **Collection date** (day/month/year) | **Latitude** | **Longitude** | **Mitochondrial haplotype**  (sequence origin) | **STRUCTURE cluster/**  **DAPC cluster** |
| --- | --- | --- | --- | --- | --- |
| A03 | 20/04/2018 | -33.98804 | 18.43421 | H2 (ref 1) | P2/P2 |
| A04 | 05/06/2018 | -34.02121 | 18.42648 | H2 (ref 1) | P2/P2 |
| A08 | 02/02/2019 | -34.02001 | 18.44015 | H2 (ref 1) | n.a. |
| A09 | 02/02/2019 | -34.01021 | 18.43829 | H2 (ref 1) | P2/P2 |
| A10 | 01/04/2016 | -34.01288 | 18.36394 | H2 (ref 1, 2) | P2/P2 |
| A11 | 28/02/2019 | -34.00375 | 18.43701 | H1 (ref 1) | P2/P2 |
| A12 | 09/05/2016 | -33.99503 | 18.41894 | H2 (ref 1, 2) | P2/P2 |
| A17 | 28/02/2019 | -33.99732 | 18.42648 | H1 (ref 1) | P2/P2 |
| A18 | NA/NA/2014 | -33.9916 | 18.42878 | H1 (ref 1, 2) | P2/P2 |
| A19 | 03/04/2019 | -33.98096 | 18.43803 | H2 (ref 1) | P2/P2 |
| A20 | NA/03/2019 | -34.00052 | 18.42716 | H2 (ref 1) | P2/P2 |
| A21 | 02/03/2019 | -34.01079 | 18.41787 | H2 (ref 1) | P2/P2 |
| A22 | 03/03/2019 | -33.97277 | 18.46348 | H2 (ref 1) | P2/P2 |
| A24 | 01/02/2020 | -34.15379 | 18.41755 | H2 (ref 1) | P2/P2 |
| A25 | NA/06/2019 | -34.12554 | 18.43230 | H2 (ref 1) | P2/P2 |
| A40 | 13/03/2021 | -33.98287 | 18.45010 | H2 (this study) | P2/P2 |
| A47 | NA/NA/2022 | -34.01927 | 18.45813 | H2 (this study) | P2/P2 |
| D02 | 18/08/2016 | -33.94508 | 18.86959 | H1 (ref 1, 2) | P1/P3 |
| D12 | 22/03/2016 | -33.94276 | 18.86966 | H2 (ref 1) | P1/P1 |
| D18 | 27/03/2018 | -33.94291 | 18.84960 | H1 (ref 1) | P1/P1 |
| D19 | 19/05/2017 | -33.94676 | 18.87246 | H2 (ref 1, 2) | P1/P1 |
| D21 | 22/03/2016 | -33.9403 | 18.88884 | H1 (ref 1, 2) | P1/P1 |
| D27 | NA/04/2019 | -33.94207 | 18.87024 | H1 (ref 1) | P1/P1 |
| D03 | 12/04/2016 | -33.94322 | 18.83403 | H1 (ref 1) | P1/P1 |
| D04 | 10/03/2016 | -33.95808 | 18.82733 | H2 (ref 1, 2) | P1/P1 |
| D08 | NA/04/2016 | -33.94611 | 18.87138 | H1 (ref 1) | P1/P1 |
| D15 | 05/04/2016 | -33.93546 | 18.85498 | H1 (ref 1, 2) | P1/P1 |
| E01 | 12/03/2018 | -33.95691 | 18.91650 | H1 (ref 1, 2) | P1/P1 |
| E02 | 15/03/2018 | -33.96674 | 18.92395 | H1 (ref 1) | P1/P1 |
| E04 | 13/05/2016 | -33.96048 | 18.92275 | H1 (ref 1, 2) | P1/P1 |
| E07 | 06/04/2017 | -33.96921 | 18.93098 | H2 (ref 1) | P1/P1 |
| F03 | 05/06/2017 | -33.9134 | 18.94706 | H1 (ref 1, 2) | P1/P1 |
| G07 | 05/04/2018 | -33.91104 | 19.10741 | H1 (this study) | P1/P1 |
| H02 | 10/04/2016 | -33.64164 | 19.02380 | H1 (ref 1, 2) | P1/P1 |
| I02 | 17/04/2018 | -33.37844 | 19.30747 | H1 (ref 1) | P1/P1 |
| I04 | 17/04/2018 | -33.42303 | 19.26493 | H1 (ref 1) | P1/P1 |
| I09 | 01/04/2022 | -33.26688 | 19.25851 | H1 (this study) | P1/P1 |
| I13 | 20/04/2022 | -33.24201 | 19.28380 | H1 (this study) | P1/P1 |
| J01 | 10/03/2016 | -33.76878 | 19.51752 | H1 (ref 1, 2) | P1/P3 |
| J03 | 31/03/2022 | -33.74734 | 19.49280 | H1 (this study) | P1/P1 |
| K01 | 07/02/2018 | -34.33548 | 19.02626 | H1 (ref 1, 2) | P1/P3 |
| K04 | 20/03/2023 | -34.34714 | 19.27456 | H1 (this study) | P1/P1 |
| L11 | 18/05/2016 | -33.82778 | 18.92348 | H1 (this study) | P1/P1 |
| L03 | 18/05/2016 | -33.83479 | 18.62710 | H1 (ref 1, 2) | P1/P1 |
| L04 | 07/06/2018 | -33.86616 | 18.61395 | H1 (ref 1) | P1/P1 |
| L09 | 16/04/2019 | -33.52518 | 19.19452 | H1 (ref 1) | P1/P1 |
| L36 | NA/NA/2022 | -33.93014 | 18.69420 | H1 (this study) | P1/P1 |
| M03 | 19/03/2019 | -34.15372 | 19.02653 | H1 (ref 1) | P1/P1 |

Eloff, J., Veldtman, R., Bulgarella, M. et al. (2020) Population genetics of the invasive wasp *Vespula germanica* in South Africa. Insect. Soc. 67, 229–238 . <https://doi.org/10.1007/s00040-020-00752-x>
van Asch, B., Wolf, M., Marais, I., Daly, D., & Veldtman, R. (2022). Mitogenomics and the global dispersion of *Vespula germanica*: A case study from South Africa shows evidence for two separate invasion events. *Diversity*, *14*(3), 154. https://doi.org/10.3390/d14030154

**Table S2.** Quality control and processing metrics obtained with FastQC for 2b-RAD sequencing data of 50 individuals of *Vespula germanica* collected in South Africa.

| **Sampled colonies** | **Raw reads** | **PEAR reads** | **Valid RAD tags** | **Clean reads** | **%** |
| --- | --- | --- | --- | --- | --- |
| A03 | 5596370 | 5307261 | 4208482 | 4007832 | 71.61 |
| A04 | 4848205 | - | 2018239 | 1887686 | 38.94 |
| A08 | 5020923 | - | 3129223 | 2928297 | 58.32 |
| A09 | 4572691 | - | 2377184 | 2223452 | 48.62 |
| A10 | 5504122 | 5289986 | 4194798 | 4027568 | 73.17 |
| A11 | 4750763 | - | 4372783 | 4164102 | 87.65 |
| A12 | 5596370 | 5307261 | 5152915 | 5149253 | 92.01 |
| A17 | 4921690 | - | 3597688 | 3429587 | 69.68 |
| A18 | 4636348 | - | 4357417 | 4164590 | 89.82 |
| A19 | 4935618 | - | 2924434 | 2741209 | 55.54 |
| A20 | 4902080 | - | 2219769 | 2075695 | 42.34 |
| A21 | 6188799 | - | 3502072 | 3278393 | 52.97 |
| A22 | 5193451 | - | 2179025 | 2029684 | 39.08 |
| A24 | 5596370 | 5307261 | 4918638 | 4915212 | 87.83 |
| A25 | 5596370 | 5307261 | 4805240 | 4801237 | 85.79 |
| A40 | 7602092 | - | 6786649 | 6420557 | 84.46 |
| A47 | 5504122 | 5289986 | 5046244 | 4790115 | 87.03 |
| D02 | 4625427 | - | 2203951 | 2105085 | 45.51 |
| D12 | 5280197 | - | 2821853 | 2642707 | 50.05 |
| D18 | 5360338 | 5175860 | 4882618 | 4623022 | 86.24 |
| D19 | 5111910 | - | 4230360 | 4037494 | 78.98 |
| D21 | 4571131 | - | 2678413 | 2505132 | 54.80 |
| D27 | 5504122 | 5289986 | 4664597 | 4660230 | 84.67 |
| D3A | 8152320 | - | 6245548 | 5914920 | 72.56 |
| D04 | 5504122 | 5289986 | 5042448 | 5038269 | 91.54 |
| D08 | 5504122 | 5289986 | 4545741 | 4541980 | 82.52 |
| D15 | 4953024 | - | 2278478 | 2170385 | 43.82 |
| E01 | 4866776 | - | 2939466 | 2811321 | 57.77 |
| E02 | 4888039 | - | 1350916 | 1265915 | 25.90 |
| E04 | 4889931 | - | 957444 | 894078 | 18.28 |
| E07 | 7266362 | - | 3729722 | 3532900 | 48.62 |
| F03 | 5360338 | 5175860 | 4908288 | 4904041 | 91.49 |
| G07 | 5360338 | 5175860 | 4980328 | 4975405 | 92.82 |
| H02 | 5234065 | - | 3219345 | 3015459 | 57.61 |
| I02 | 5543486 | 5252598 | 4323646 | 4087211 | 73.73 |
| I04 | 5543486 | 5252598 | 4955032 | 4949986 | 89.29 |
| I09 | 5543486 | 5252598 | 3444338 | 3440910 | 62.07 |
| I13 | 5543486 | 5252598 | 4220690 | 4215976 | 76.05 |
| J01 | 5057610 | - | 3374463 | 3247032 | 64.20 |
| J03 | 5543486 | 5252598 | 4927080 | 4701631 | 84.81 |
| K01 | 4371970 | - | 3459660 | 3305885 | 75.62 |
| K04 | 6118430 | 5934946 | 5318641 | 5036067 | 82.31 |
| L11 | 6118430 | 5934946 | 5673504 | 5422236 | 88.62 |
| L03 | 6118430 | 5934946 | 5672072 | 5666868 | 92.62 |
| L04 | 6118430 | 5934946 | 5086036 | 5081357 | 83.05 |
| L09 | 6118430 | 5934946 | 5687093 | 5681171 | 92.85 |
| L36 | 4833300 | - | 4571849 | 4270520 | 88.36 |
| M03 | 6232079 | - | 4283923 | 4010366 | 64.35 |

**Table S3.** Description of environmental and distance variables used in the Generalised Additive Model (GAM) analysis of genetic differentiation in invasive *Vespula germanica* in South Africa.

| **Category** | **Variable name** | **Description** | **Temporal scale** |
| --- | --- | --- | --- |
| **Precipitation** | rmean | Mean annual rainfall (mm/year) | 30-year average |
| **Temperature** | tmaxave | Mean annual maximum temperature (°C) | 30-year average |
|  | tmean | Mean annual temperature (°C) | 30-year average |
|  | tmaxsum | Mean summer maximum temperature (Jan-Mar) (°C) | 30-year average |
|  | tminwint | Mean winter minimum temperature (°C) | 30-year average |
|  | tminave | Mean annual minimum temperature (°C) | 30-year average |
| **Landscape moisture** | NDMI16 | Normalized Difference Moisture Index (2016) | Single year (2016) |
|  | NDM19 | Normalized Difference Moisture Index (2019) | Single year (2019) |
| **Distance** | dist_geo_km | Distance between both individual |  |
|  | dist_km_CT | Distance from the Cape Town invasion core |  |
|  | dist_km_SB | Distance from the Stellenbosch invasion core |  |

**Table S4.** **Results of the final shape-constrained additive model (SCAM) of Vespula germanica sampled in South Africa (n = 47, 1081 pairs, after exclusion of two outliers males).** The model uses a quasi-binomial family (logit link) with shape constraints: “miso” (monotonically increasing from zero) for geographical distance between individuals. Non-significant environmental terms were excluded. R-sq.(adj) = 0.560; explained deviance = 56.3%; GCV = 0.00262; n = 1081.

| Component | Variable | Estimate (Std. Error) | Statistic | p-value | Significance |
| --- | --- | --- | --- | --- | --- |
| Parametric terms | Intercept | -0.517 (0.010) | t = -49.4 | < 2e-16 | *** |
|  | Δ Distance to Stellenbosch (km) | -0.00070 (0.00021) | t = -3.32 | 0.00092 | *** |
|  | SNP cluster pair P1^+3^-P2 | 0.172 (0.008) | t = 22.0 | < 2e-16 | *** |
|  | SNP cluster pair P2-P2 | -0.078 (0.013) | t = -6.20 | 8.10e-10 | *** |
| Smooth terms | Geographic distance between individuals (monotonic increasing from zero) | edf = 2.56 | F = 26.6 (Ref.df = 3.12) | < 2e-16 | *** |

**Table S5. Hierarchical variance partitioning of genetic differentiation (*F_ST_*) in Vespula germanica in South Africa (n = 47, 1081 pairs, excluding two male outliers).** Contributions are based on deviance explained from reduced SCAM models (quasibinomial, logit link). The negative shared-all contribution is interpreted as zero due to high multicollinearity among spatial, environmental, and genetic factors. Total deviance explained = 56.3%.

| Component | Absolute Contribution | Relative Contribution (%) | 1000 Permutation p-value* |
| --- | --- | --- | --- |
| Unique Spatial | 0.017 | 3.0 | 0.001 |
| Unique Environmental | 0.000 | 0.0 | 0.989 |
| Unique Genetic | 0.163 | 28.9 | 0.006 |
| Shared Spatial-Environmental | 0.220 | 39.0 | NA |
| Shared Spatial-Genetic | 0.314 | 55.7 | NA |
| Shared Environmental-Genetic | 0.255 | 45.3 | NA |
| Shared All | 0.000 | 0.0 | NA |

* Empirical p-values were calculated as 𝑝 = ( # { Δ 𝑅 perm 2 ≥ Δ 𝑅 observed 2 } + 1 ) / ( 𝑁 success + 1 ) p=(#{ΔR perm 2 ​ ≥ΔR observed 2 ​ }+1)/(N success ​ +1), where 𝑁 success N success ​ is the number of successful model fits.

**Table S6.** Coordinates and average values of environmental variables measured for each geographical point of each sampled colony (rainfall, temperature, moisture). See csv file. [SEE corresponding CSV FILE]


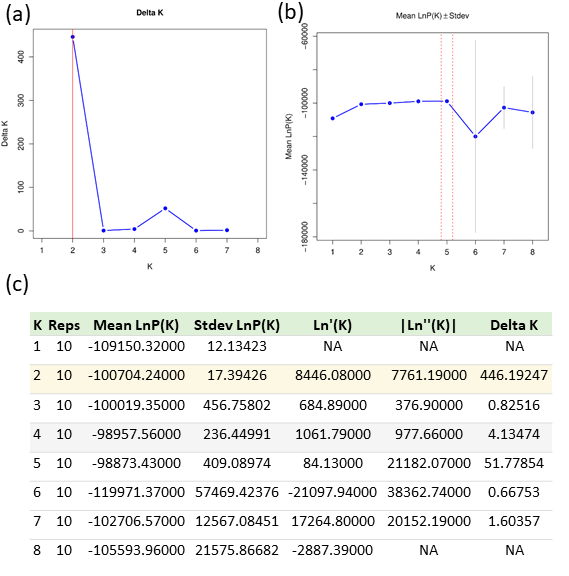


**Figure S1. Selection of the most likely number of genetic clusters (K) inferred by STRUCTURE analysis**. (a) Delta K method (Evanno et al. 2005), (b) Mean log-likelihood of the data [LnP(K)] ± standard deviation across 10 replicates for each K value, (c) Summary table of STRUCTURE results showing Mean LnP(K), its standard deviation, first and second-order rate of change (Ln′(K), Ln′′(K)), and Delta K values.

**
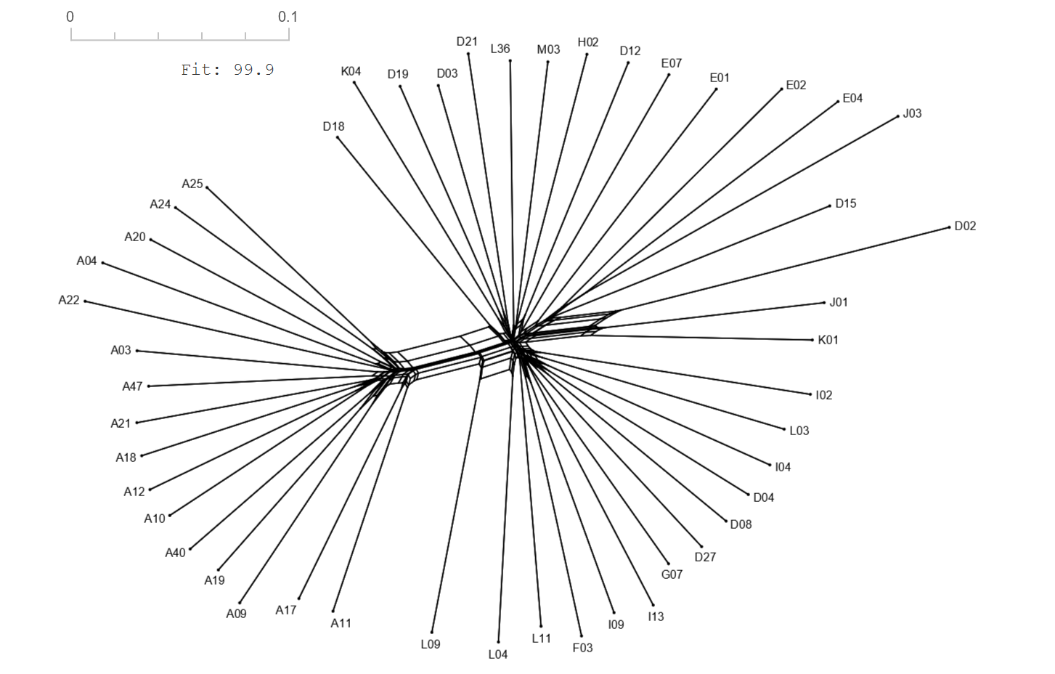
**

**Figure S2. Neighbor-Net phylogenetic tree of *V. germanica* collected in South Africa, based on genome-wide SNP data for 49 individuals (4,910 sites).** The network was made on SplitsTree using the “Handle Ambiguous States = Average States" option and achieves a Fit of 99.9%. The 129 splits, visualized as parallel or intersecting lines show reticulations and conflicting phylogenetic signals**.** The scale bar represents genetic distance.


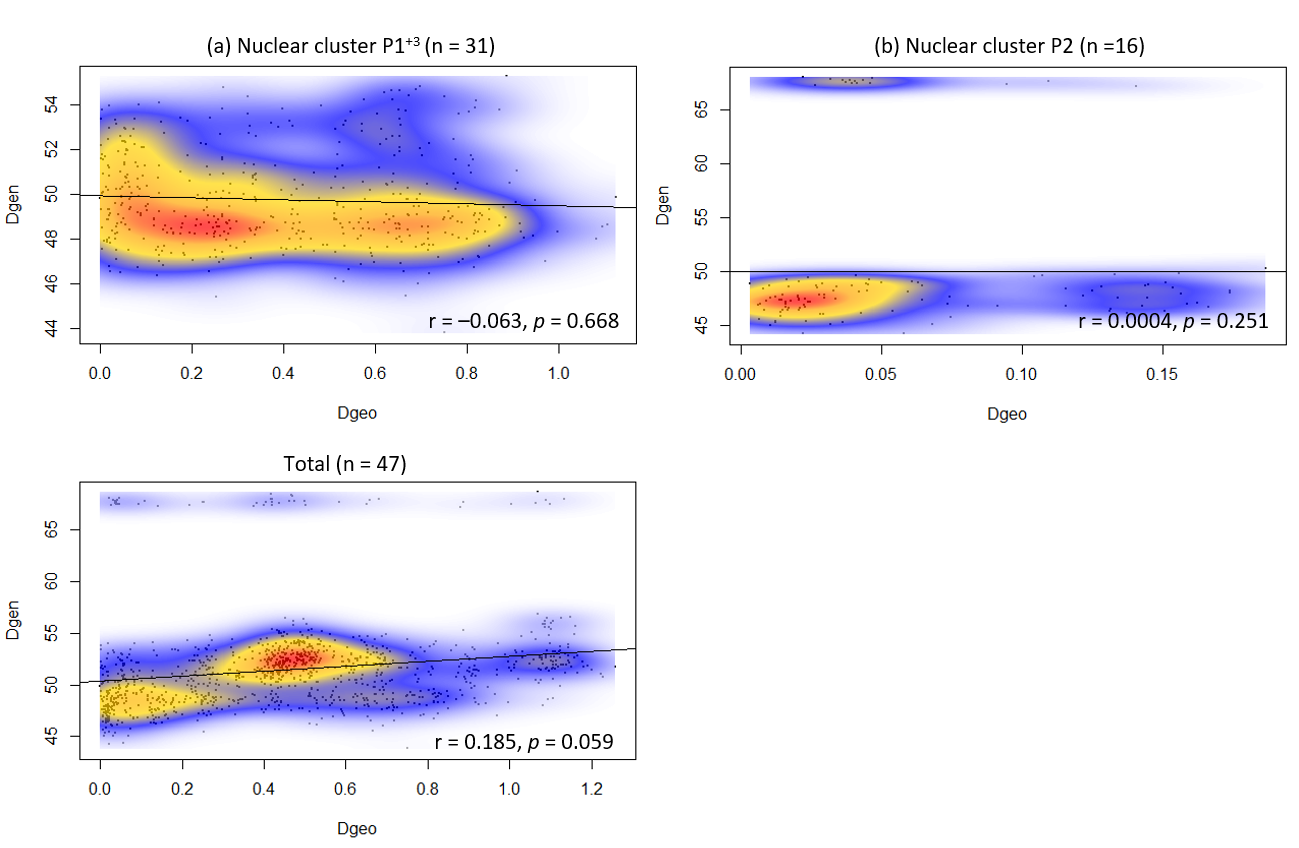


**Figure S3. Kernel density plots of the relationship between geographic and genetic Euclidian distances for the nuclear SNP clusters (a) P1^+3^, (b) P2, and (c) for the full dataset.** Density shading represents point concentration, with fitted regression lines shown in red. The plots were generated with the MASS package. Results of the Mantel tests with 10,000 permutations are indicated in each panel.
