# Supplementary material for: Genomic Evidence for Dual Introductions, Limited Gene Flow and Niche Preferences in the Invasive Wasp Vespula germanica in South Africa
Source: Mol Ecol. 2025 Dec 22;35(1):e70217. doi: 10.1111/mec.70217 (PMC12745851; doi:10.1111/mec.70217)

Analysis of genetic differentiation and climatic variation in *Vespula germanica*:

SCAM_hierarchical_variables_climatic_comparisons.R

```
> library(caret)
> library(mgcv)
> library(scam)
> library(dplyr)
> library(ggplot2)
> library(sf)
> library(rnaturalearth)
> library(gratia)
> library(caret)
> library(dplyr)
> library(tidyr)
> library(dplyr)
> library(FSA)
> library(ggpubr)

# SCAM Analysis for genetic differentiation in Vespula germanica (No Males)
# Description: Fits shape-constrained additive models (SCAM) to model FST, performs hierarc
hical variance partitioning, and conducts residual diagnostics for 47 individuals (1081 pai
rs).

# ---- Data Preparation ----
# Load and merge datasets
> data <- read.csv("Comparison_data.csv", sep = ";", header = TRUE)
> coords <- read.csv("coordinates_47.csv", sep = ";", header = TRUE)
# Merge pairwise data with coordinates and compute mean coordinates
> data <- data %>%
+   left_join(coords, by = c("ind1" = "ind")) %>%
+   left_join(coords, by = c("ind2" = "ind"), suffix = c("_ind1", "_ind2")) %>%
+   mutate(
+     x = (lon_ind1 + lon_ind2) / 2,
+     y = (lat_ind1 + lat_ind2) / 2
+   )
# Check correlations between predictors (columns 4:6, 9:17)
> corr_mat <- cor(data[, c(4:6, 9:17)])
> print(corr_mat)
                 dist_geo_km delta_dist_km_CT delta_dist_km_SB        fst delta_tmaxave del
ta_tmean  delta_rmean delta_NDMI16 delta_NDMI19 delta_tmaxsum delta_tminwint
dist_geo_km        1.0000000        0.9151974       0.46437482 0.49359317     0.4774480  0.
55611977  0.243065868   0.28682492   0.28985691    0.44939021      0.7586365
delta_dist_km_CT   0.9151974        1.0000000       0.43178617 0.53455884     0.3951883  0.
40400601  0.367249828   0.26977626   0.29220299    0.39719433      0.7009395
delta_dist_km_SB   0.4643748        0.4317862       1.00000000 0.20974635     0.4136024  0.
37816392  0.093578010   0.14020470   0.09157691    0.33810917      0.3722393
fst                0.4935932        0.5345588       0.20974635 1.00000000     0.3734661  0.
09131826  0.325819411   0.08099572   0.13301363    0.44035112      0.2324430
delta_tmaxave      0.4774480        0.3951883       0.41360240 0.37346608     1.0000000  0.
51625550  0.302107318   0.12201210   0.15029796    0.94451201      0.1890032
delta_tmean        0.5561198        0.4040060       0.37816392 0.09131826     0.5162555  1.
00000000 -0.009297260   0.28658403   0.27956789    0.30833475      0.6134944
delta_rmean        0.2430659        0.3672498       0.09357801 0.32581941     0.3021073 -0.
00929726  1.000000000   0.13189100   0.17019681    0.29433364      0.0465475
delta_NDMI16       0.2868249        0.2697763       0.14020470 0.08099572     0.1220121  0.
28658403  0.131891001   1.00000000   0.80133805    0.05927132      0.3708321
delta_NDMI19       0.2898569        0.2922030       0.09157691 0.13301363     0.1502980  0.
27956789  0.170196808   0.80133805   1.00000000    0.11376392      0.3412730
delta_tmaxsum      0.4493902        0.3971943       0.33810917 0.44035112     0.9445120  0.
30833475  0.294333644   0.05927132   0.11376392    1.00000000      0.1223940
delta_tminwint     0.7586365        0.7009395       0.37223935 0.23244303     0.1890032  0.
61349438  0.046547497   0.37083207   0.34127301    0.12239397      1.0000000
```

```
delta_tminave      0.6769087        0.6120416       0.34262502 0.22401947     0.1486068  0.
65300889  0.006716299   0.37562127   0.35878890    0.06756771      0.9519128
                 delta_tminave
dist_geo_km        0.676908733
delta_dist_km_CT   0.612041594
delta_dist_km_SB   0.342625016
fst                0.224019473
delta_tmaxave      0.148606842
delta_tmean        0.653008895
delta_rmean        0.006716299
delta_NDMI16       0.375621273
delta_NDMI19       0.358788897
delta_tmaxsum      0.067567714
delta_tminwint     0.951912764
delta_tminave      1.000000000

# ---- Full SCAM Model ----
# Fit full SCAM model with spatial, environmental, and genetic predictors kept after checki
ng correlation
> model_full <- scam(
+   fst ~ s(dist_geo_km, bs = "miso") +  # Monotonic increasing geographic distance
+     delta_dist_km_SB +                 # Linear distance to Stellenbosch
+     s(delta_tmean, bs = "mpi") +       # Monotonic positive mean temperature difference
+     s(delta_rmean, bs = "mpi") +       # Monotonic positive precipitation difference
+     s(delta_NDMI16, bs = "mpi") +      # Monotonic positive NDMI difference
+     s(delta_tmaxsum, bs = "mpi") +     # Monotonic positive max summer temperature differ
ence
+     clus_type,                         # Genetic cluster type
+   data = data,
+   family = quasibinomial(link = "logit"),
+   optimizer = "efs"
+ )
# Summarize and check model diagnostics
> summary(model_full)

Family: quasibinomial
Link function: logit

Formula:
fst ~ s(dist_geo_km, bs = "miso") + delta_dist_km_SB + s(delta_tmean,
    bs = "mpi") + s(delta_rmean, bs = "mpi") + s(delta_NDMI16,
    bs = "mpi") + s(delta_tmaxsum, bs = "mpi") + clus_type

Parametric coefficients:
                   Estimate Std. Error t value Pr(>|t|)
(Intercept)      -0.5165401  0.0104490 -49.434  < 2e-16 ***
delta_dist_km_SB -0.0006960  0.0002096  -3.320 0.000929 ***
clus_typeP1P2     0.1718591  0.0077971  22.042  < 2e-16 ***
clus_typeP2      -0.0782774  0.0126291  -6.198 8.13e-10 ***
---
Signif. codes:  0 ‘***’ 0.001 ‘**’ 0.01 ‘*’ 0.05 ‘.’ 0.1 ‘ ’ 1

Approximate significance of smooth terms:
                   edf Ref.df     F p-value
s(dist_geo_km)   2.553  3.114 26.61  <2e-16 ***
s(delta_tmean)   0.000  0.000    NA      NA
s(delta_rmean)   0.000  0.000    NA      NA
s(delta_NDMI16)  0.000  0.000    NA      NA
s(delta_tmaxsum) 0.000  0.000    NA      NA
---
Signif. codes:  0 ‘***’ 0.001 ‘**’ 0.01 ‘*’ 0.05 ‘.’ 0.1 ‘ ’ 1

R-sq.(adj) =  0.5599   Deviance explained = 56.3%
GCV score = 0.002619  Scale est. = 0.0026031  n = 1081

> appraise(model_full)
```

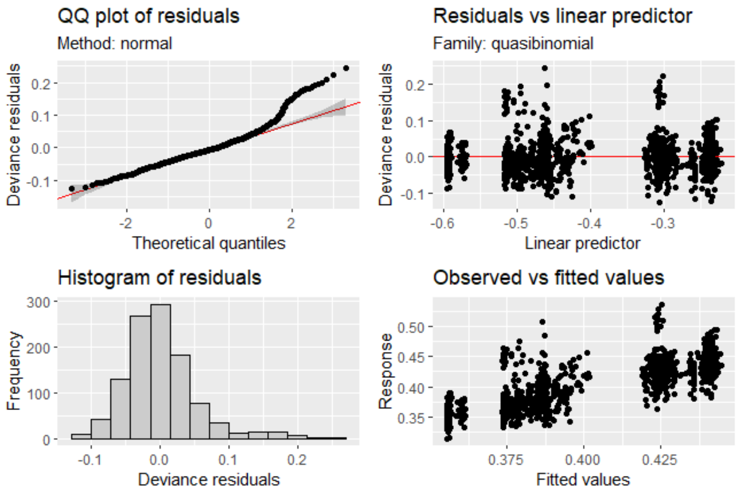


```
> deviance(model_full)
[1] 2.796895
# ---- Simplified SCAM Model ----
# Fit simplified SCAM model (removing non-significant environmental terms)
> model_simp <- scam(
+   fst ~ s(dist_geo_km, bs = "miso") +  # Monotonic increasing geographic distance
+     delta_dist_km_SB +                 # Linear distance to Stellenbosch
+     clus_type,                         # Genetic cluster type
+   data = data,
+   family = quasibinomial(link = "logit"),
+   optimizer = "efs"
+ )
# Summarize, check diagnostics, and plot smooth terms
> summary(model_simp)

Family: quasibinomial
Link function: logit

Formula:
fst ~ s(dist_geo_km, bs = "miso") + delta_dist_km_SB + clus_type

Parametric coefficients:
                   Estimate Std. Error t value Pr(>|t|)
(Intercept)      -0.5165054  0.0104489 -49.431  < 2e-16 ***
delta_dist_km_SB -0.0006964  0.0002096  -3.322 0.000924 ***
clus_typeP1P2     0.1718573  0.0077988  22.036  < 2e-16 ***
clus_typeP2      -0.0782819  0.0126288  -6.199  8.1e-10 ***
---
Signif. codes:  0 ‘***’ 0.001 ‘**’ 0.01 ‘*’ 0.05 ‘.’ 0.1 ‘ ’ 1

Approximate significance of smooth terms:
                 edf Ref.df     F p-value
s(dist_geo_km) 2.557  3.119 26.58  <2e-16 ***
---
Signif. codes:  0 ‘***’ 0.001 ‘**’ 0.01 ‘*’ 0.05 ‘.’ 0.1 ‘ ’ 1

R-sq.(adj) =  0.5599   Deviance explained = 56.3%
GCV score = 0.002619  Scale est. = 0.0026031  n = 1081

> appraise(model_simp)
```

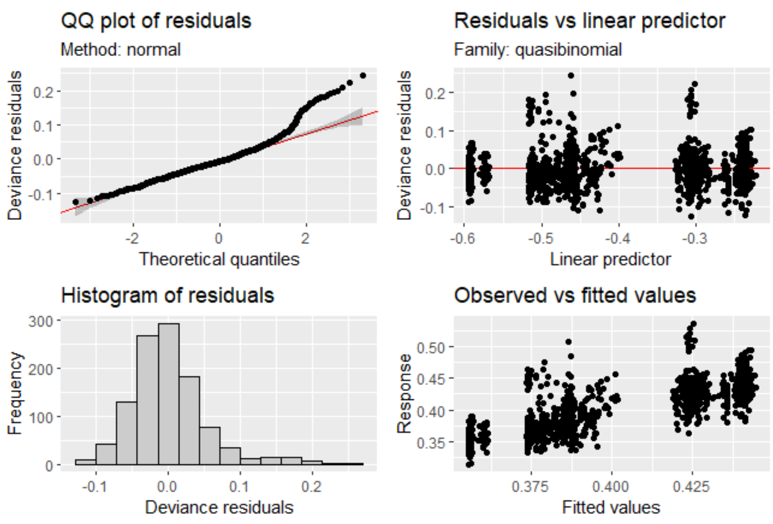


```
> deviance(model_simp)
[1] 2.796861
> plot(model_simp, shade = TRUE, main = "Smooth Term for Geographic Distance")
```

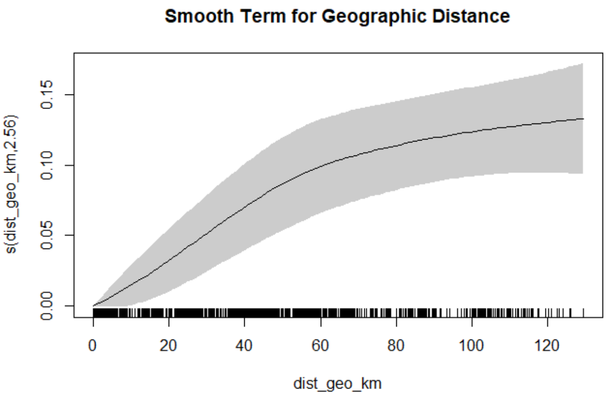


```
# Extract deviance residuals and fitted values
> resid_dev <- residuals(model_simp, type = "deviance")
> fitted_vals <- fitted(model_simp)

# Histogram of deviance residuals
> hist(resid_dev, breaks = 40, main = "Histogram of Deviance Residuals",
+      xlab = "Deviance Residuals")
```

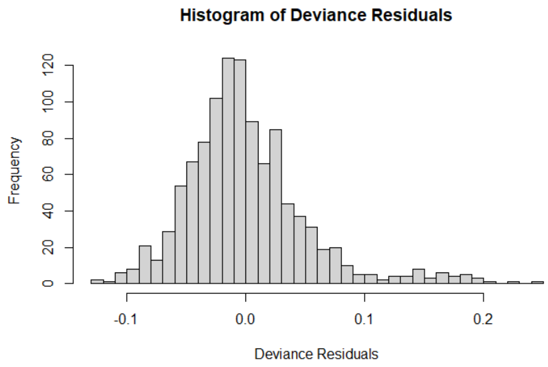


```
# Plot residuals vs key predictors
> plot(data$dist_geo_km, resid_dev, pch = 20, main = "Residuals vs Geographic Distance",
+      xlab = "Geographic Distance (km)", ylab = "Deviance Residuals")
```

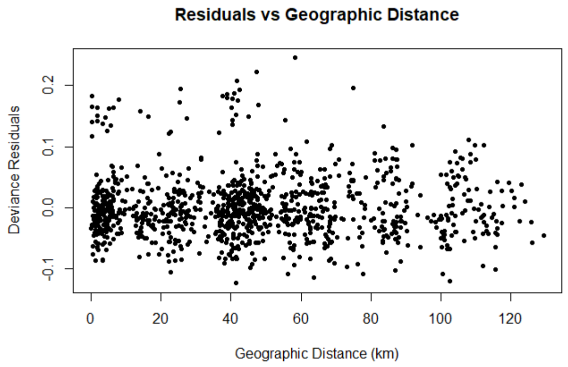


```
# Spatial pattern of residuals
> ggplot(data, aes(x = x, y = y, colour = resid_dev)) +
+   geom_point() +
+   scale_colour_gradient2(midpoint = 0, low = "blue", mid = "white", high = "red") +
+   labs(title = "Spatial Pattern of Deviance Residuals", x = "Mean Longitude", y = "Mean L
atitude") +
+   theme_minimal()
```

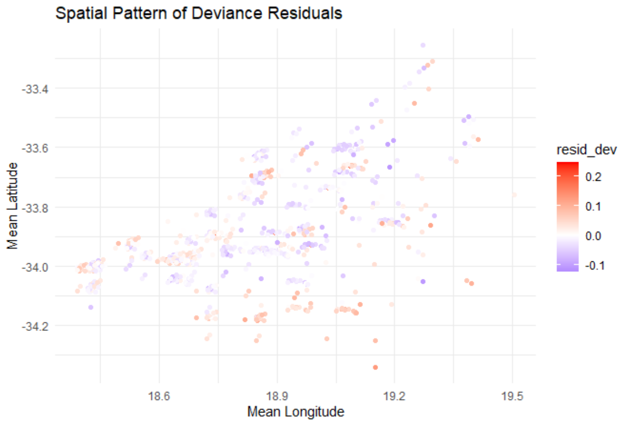


```
# ---- Hierarchical Variance Partitioning ----
# Extract deviance explained from full model
> R2_full<- summary(model_full)$dev.expl
# Fit reduced models for partitioning
> model_spatial <- scam(
+   fst ~ s(dist_geo_km, bs = "miso") + delta_dist_km_SB,
+   data = data,
+   family = quasibinomial(link = "logit"),
+   optimizer = "efs"
+ )
> R2_spatial <- summary(model_spatial)$dev.expl
> model_env <- scam(
+   fst ~ s(delta_tmean, bs = "mpi") + s(delta_rmean, bs = "mpi") +
+     s(delta_NDMI16, bs = "mpi") + s(delta_tmaxsum, bs = "mpi"),
+   data = data,
+   family = quasibinomial(link = "logit"),
+   optimizer = "efs"
+ )
> R2_env <- summary(model_env)$dev.expl
> model_gen <- scam(
+   fst ~ clus_type,
+   data = data,
+   family = quasibinomial(link = "logit"),
+   optimizer = "efs"
+ )
```

```
> R2_gen <- summary(model_gen)$dev.expl
# Fit models excluding one group
> model_without_spatial <- scam(
+   fst ~ s(delta_tmean, bs = "mpi") + s(delta_rmean, bs = "mpi") +
+     s(delta_NDMI16, bs = "mpi") + s(delta_tmaxsum, bs = "mpi") + clus_type,
+   data = data,
+   family = quasibinomial(link = "logit"),
+   optimizer = "efs"
+ )
> R2_without_spatial <- summary(model_without_spatial)$dev.expl
> model_without_env <- scam(
+   fst ~ s(dist_geo_km, bs = "miso") + delta_dist_km_SB + clus_type,
+   data = data,
+   family = quasibinomial(link = "logit"),
+   optimizer = "efs"
+ )
> R2_without_env <- summary(model_without_env)$dev.expl
> model_without_gen <- scam(
+   fst ~ s(dist_geo_km, bs = "miso") + delta_dist_km_SB +
+     s(delta_tmean, bs = "mpi") + s(delta_rmean, bs = "mpi") +
+     s(delta_NDMI16, bs = "mpi") + s(delta_tmaxsum, bs = "mpi"),
+   data = data,
+   family = quasibinomial(link = "logit"),
+   optimizer = "efs"
+ )
> R2_without_gen <- summary(model_without_gen)$dev.expl
# Calculate unique and shared contributions
> unique_spatial <- R2_full - R2_without_spatial
> unique_env <- R2_full - R2_without_env
> unique_gen <- R2_full - R2_without_gen
> shared_spatial_env <- R2_spatial + R2_env - R2_without_gen
> shared_spatial_gen <- R2_spatial + R2_gen - R2_without_env
> shared_env_gen <- R2_env + R2_gen - R2_without_spatial
> shared_all <- R2_full - (unique_spatial + unique_env + unique_gen +
+                                      shared_spatial_env + shared_spatial_gen + shared_env_g
en)
# Calculate relative contributions (% of R2_full)
> rel_unique_spatial <- (unique_spatial / R2_full) * 100
> rel_unique_env <- (unique_env / R2_full) * 100
> rel_unique_gen <- (unique_gen / R2_full) * 100
> rel_shared_spatial_env <- (shared_spatial_env / R2_full) * 100
> rel_shared_spatial_gen <- (shared_spatial_gen / R2_full) * 100
> rel_shared_env_gen <- (shared_env_gen / R2_full) * 100
> rel_shared_all <- (shared_all / R2_full) * 100
# Summarize results in a data frame
> results_varpart <- data.frame(
+   Component = c("Unique Spatial", "Unique Environmental", "Unique Genetic",
+                 "Shared Spatial-Environmental", "Shared Spatial-Genetic",
+                 "Shared Environmental-Genetic", "Shared All"),
+   Contribution = c(unique_spatial, unique_env, unique_gen,
+                    shared_spatial_env, shared_spatial_gen, shared_env_gen, shared_all),
+   Relative_Percent = c(rel_unique_spatial, rel_unique_env, rel_unique_gen,
+                        rel_shared_spatial_env, rel_shared_spatial_gen,
+                        rel_shared_env_gen, rel_shared_all)
+ )
# Print and verify total contribution
> cat("Variance Partitioning Results:\n")
Variance Partitioning Results:
> print(results_varpart)
                     Component  Contribution Relative_Percent
1               Unique Spatial  1.678879e-02     2.980562e+00
2         Unique Environmental -5.341348e-06    -9.482649e-04
3               Unique Genetic  1.629263e-01     2.892478e+01
4 Shared Spatial-Environmental  2.195285e-01     3.897352e+01
5       Shared Spatial-Genetic  3.136364e-01     5.568078e+01
6 Shared Environmental-Genetic  2.550469e-01     4.527922e+01
```

```
7                   Shared All -4.046457e-01    -7.183792e+01
> cat("Sum of relative contributions (%):", sum(results_varpart$Relative_Percent), "\n")
Sum of relative contributions (%): 100


# --- Permutation test ---
#To assess whether removing a block of predictors (spatial/environmental/genetic)
#significantly reduces the model's explanation (ΔR²). This allows the statistical
#importance of each component to be estimated.
> perm_test_models <- function(form_full, form_reduced, data, N_perm = 1000, seed = 123,
+                              family = quasibinomial(link = "logit"), optimizer = "efs",
+                              two_sided = FALSE, verbose = TRUE) {
+   set.seed(seed)
+   # Observed statistic
+   m_full_obs <- scam(form_full, data = data, family = family, optimizer = optimizer)
+   R2_full_obs <- summary(m_full_obs)$dev.expl
+   m_red_obs <- scam(form_reduced, data = data, family = family, optimizer = optimizer)
+   R2_red_obs <- summary(m_red_obs)$dev.expl
+   obs_stat <- R2_full_obs - R2_red_obs
+
+   perm_stats <- rep(NA_real_, N_perm)
+   if (verbose) pb <- txtProgressBar(min = 0, max = N_perm, style = 3)
+
+   for (i in seq_len(N_perm)) {
+     data_perm <- data
+     data_perm$fst <- sample(data_perm$fst)
+
+     perm_stat_i <- tryCatch({
+       m_full_p <- scam(form_full, data = data_perm, family = family, optimizer = optimize
r)
+       R2_full_p <- summary(m_full_p)$dev.expl
+
+       m_red_p <- scam(form_reduced, data = data_perm, family = family, optimizer = optimi
zer)
+       R2_red_p <- summary(m_red_p)$dev.expl
+
+       R2_full_p - R2_red_p
+     }, error = function(e) {
+       NA_real_
+     })
+
+     perm_stats[i] <- perm_stat_i
+     if (verbose) setTxtProgressBar(pb, i)
+   }
+   if (verbose) close(pb)
+
+   perm_ok <- perm_stats[!is.na(perm_stats)]
+   n_success <- length(perm_ok)
+   if (n_success == 0) stop("Aucune permutation n'a abouti. Vérifier les modèles / données
.")
+
+   if (two_sided) {
+     p_value <- (sum(abs(perm_ok) >= abs(obs_stat)) + 1) / (n_success + 1)
+   } else {
+     p_value <- (sum(perm_ok >= obs_stat) + 1) / (n_success + 1)
+   }
+
+   res <- list(obs_stat = obs_stat, perm_stats = perm_ok, n_success = n_success, p_value =
p_value)
+   if (verbose) {
+     cat("Observed ΔR²:", obs_stat, "\n")
+     cat("Successful permutations:", n_success, "/", N_perm, "\n")
+     cat("Permutation p-value:", p_value, ifelse(two_sided, " (two-sided)\n", " (one-sided
obs>=perm)\n"))
+     hist(perm_ok, breaks = 40, main = "Null dist. of permuted ΔR²", xlab = "ΔR² (perm)")
+     abline(v = obs_stat, col = "red", lwd = 2)
```

```
+   }
+   return(res)
+ }
> # ---- Define formulas for tests ----
> form_full <- formula(fst ~ s(dist_geo_km, bs = "miso") + delta_dist_km_SB +
+                        s(delta_tmean, bs = "mpi") + s(delta_rmean, bs = "mpi") +
+                        s(delta_NDMI16, bs = "mpi") + s(delta_tmaxsum, bs = "mpi") +
+                        clus_type)
> form_no_spatial <- formula(fst ~ s(delta_tmean, bs = "mpi") + s(delta_rmean, bs = "mpi")
+
+                              s(delta_NDMI16, bs = "mpi") + s(delta_tmaxsum, bs = "mpi") +
clus_type)
> form_no_env <- formula(fst ~ s(dist_geo_km, bs = "miso") + delta_dist_km_SB + clus_type)
> form_no_gen <- formula(fst ~ s(dist_geo_km, bs = "miso") + delta_dist_km_SB +
+                          s(delta_tmean, bs = "mpi") + s(delta_rmean, bs = "mpi") +
+                          s(delta_NDMI16, bs = "mpi") + s(delta_tmaxsum, bs = "mpi"))
# ---- Run permutation tests (adjust N_perm if needed) ----
> N_perm_global <- 1000   # modifier ici si tu veux plus de permutations (ex: 10000)
> cat("Running permutation tests (this may take time): N_perm =", N_perm_global, "\n")
Running permutation tests (this may take time): N_perm = 1000
res_spatial <- perm_test_models(form_full, form_no_spatial, data, N_perm = N_perm_global, s
eed = 123)
  |===========================================================================================
===========| 100%
Observed ΔR²: 0.01678879
Successful permutations: 998 / 1000
Permutation p-value: 0.001001001  (one-sided obs>=perm)
```

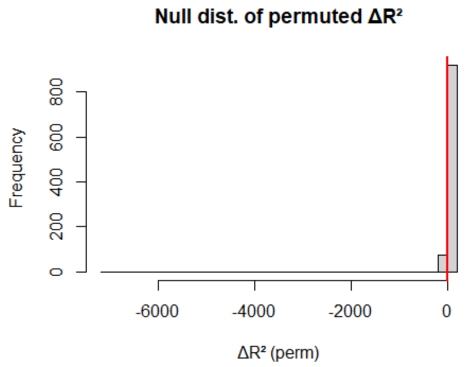


```
> res_env     <- perm_test_models(form_full, form_no_env,     data, N_perm = N_perm_global,
seed = 123)
|=============================================================================================
=========| 100%
Observed ΔR²: -5.341348e-06
Successful permutations: 998 / 1000
Permutation p-value: 0.98999  (one-sided obs>=perm)
```

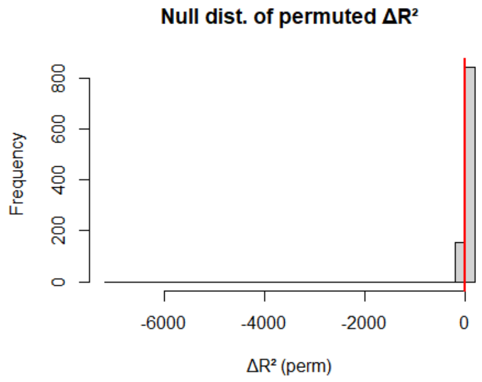

```
  |=========================================================================================
===========| 100%
Observed ΔR²: 0.1629263
Successful permutations: 995 / 1000
Permutation p-value: 0.006024096  (one-sided obs>=perm)
```

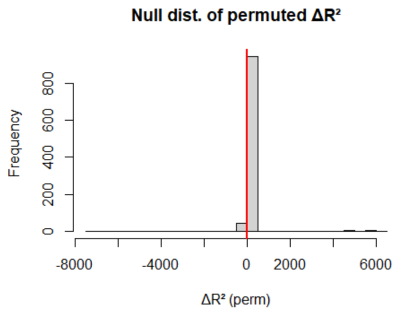


```
# ---- Report results succinctly ----
> cat("\nPermutation test results summary:\n")

Permutation test results summary:
> cat(sprintf("Spatial:    ΔR² = %.6f, permutations succeeded = %d, p = %.5f\n",
+             res_spatial$obs_stat, res_spatial$n_success, res_spatial$p_value))
Spatial:    ΔR² = 0.016789, permutations succeeded = 998, p = 0.00100
> cat(sprintf("Environmental: ΔR² = %.6f, permutations succeeded = %d, p = %.5f\n",
+             res_env$obs_stat, res_env$n_success, res_env$p_value))
Environmental: ΔR² = -0.000005, permutations succeeded = 998, p = 0.98999
> cat(sprintf("Genetic:    ΔR² = %.6f, permutations succeeded = %d, p = %.5f\n",
+             res_gen$obs_stat, res_gen$n_success, res_gen$p_value))
Genetic:    ΔR² = 0.162926, permutations succeeded = 995, p = 0.00602



# Kruskal-Wallis and Dunn's Test for FST comparison across SNP Cluster pairs
# Description: Performs a Kruskal-Wallis test to compare FST across SNP cluster pairs
# (P1P1, P1P2, P2) in Vespula germanica (n=47 individuals, 1081 pairs, no males),
# followed by Dunn's post-hoc test with Bonferroni correction.

> data$clus_type <- as.factor(data$clus_type)
> # Check normality per cluster pair using Shapiro-Wilk test
> shapiro_results <- data %>%
+   group_by(clus_type) %>%
+   summarise(
+     statistic = shapiro.test(fst)$statistic,
+     p.value = shapiro.test(fst)$p.value
+   )
> print(shapiro_results)
# A tibble: 3 × 3
  clus_type statistic  p.value
  <fct>         <dbl>    <dbl>
1 P1            0.928 3.82e-14
2 P1P2          0.945 1.20e-12
3 P2            0.990 5.71e- 1
> # Perform Kruskal-Wallis test
> kruskal_result <- kruskal.test(fst ~ clus_type, data = data)
> print(kruskal_result)

         Kruskal-Wallis rank sum test

data:  fst by clus_type
Kruskal-Wallis chi-squared = 603.08, df = 2, p-value < 2.2e-16

> # Perform Dunn's post-hoc test with Bonferroni correction
> dunn_result <- dunnTest(fst ~ clus_type, data = data, method = "bonferroni")
> print(dunn_result)
```

```
Dunn (1964) Kruskal-Wallis multiple comparison
  p-values adjusted with the Bonferroni method.

  Comparison          Z      P.unadj        P.adj
1  P1 - P1P2 -19.957552 1.288720e-88 3.866161e-88
2    P1 - P2   7.037647 1.955135e-12 5.865406e-12
3  P1P2 - P2  19.746378 8.618206e-87 2.585462e-86
> #Visualisation
> ggplot(data, aes(x = clus_type, y = fst, fill = clus_type)) +
+   geom_boxplot() +
+   labs(title = "FST by Cluster", x = "SNP cluster Combination", y = "FST") +
+   scale_fill_manual(
+     values = c("P1" = "#FFB300", "P1P2" = "#597900", "P2" = "#00796B")
+   ) +
+   theme_minimal()
```

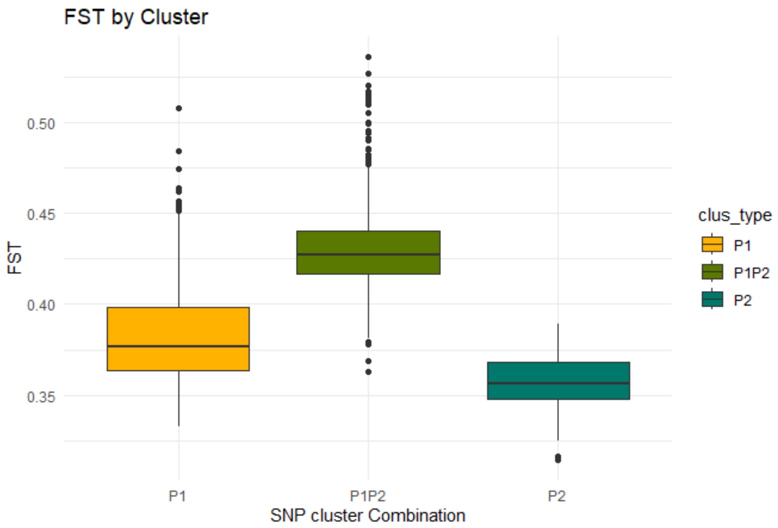


```
# Permutation test in order to assessing the climatic-environmental differences between
#genetic SNP cluster. The observed mean difference between clusters was calculated,
#followed by 10,000 permutations in which cluster labels (P1/P2) were shuffled
#while climate values remained fixed. Two-sided p-values were derived as the
#proportion of permuted differences as or more extreme than the observed


# Load data
> compar_data <- read.csv("compar_data_49.csv", sep=";", header = TRUE)
# Function to calculate difference in means
> diff_mean <- function(var, group) {
+   mean(var[group == "P1"], na.rm = TRUE) - mean(var[group == "P2"], na.rm = TRUE)
+ }
# Set seed for reproducibility
> set.seed(123)
> N_perm <- 10000
# Perform permutation tests for each climate variable
> climate_vars <- c("tmean", "rmean", "tmaxsum", "NDMI16")
> p_values <- list()
> for (var in climate_vars) {
+   # Observed difference
+   obs_diff <- diff_mean(compar_data[[var]], compar_data$structure)
+
+   # Permutations: shuffle cluster labels and recalculate differences
+   perm_diffs <- replicate(N_perm, {
+     shuffled_structure <- sample(compar_data$structure)
+     diff_mean(compar_data[[var]], shuffled_structure)
+   })
```

```
+
+   # Two-sided p-value: proportion of permuted differences as extreme as observed
+   p_values[[var]] <- mean(abs(perm_diffs) >= abs(obs_diff))
+ }
> cat("Permutation test p-values:\n")
Permutation test p-values:
> cat(sprintf("rmean: %.4f\n", p_values$rmean))
rmean: 0.0000
> cat(sprintf("tmean: %.4f\n", p_values$tmean))
tmean: 0.5045
> cat(sprintf("tmaxsum: %.4f\n", p_values$tmaxsum))
tmaxsum: 0.0000
> cat(sprintf("NDMI16: %.4f\n", p_values$NDMI16))
NDMI16: 0.0017

# Visualisation

# prepare environmental data
> envir_data <- compar_data %>%
+   select(ind, haplotype, structure, tmean, rmean, NDMI16, tmaxsum) %>%
+   pivot_longer(cols = c(tmean, rmean, NDMI16, tmaxsum),
+                names_to = "climate_variable",
+                values_to = "value")
> # Filter data and annotations for each climate variable
> data_rmean <- envir_data %>% filter(climate_variable == "rmean")
> data_tmean <- envir_data %>% filter(climate_variable == "tmean")
> data_tmaxsum <- envir_data %>% filter(climate_variable == "tmaxsum")
> data_NDMI16 <- envir_data %>% filter(climate_variable == "NDMI16")
> # Calculate y-positions for significance stars
> ranges <- envir_data %>%
+   group_by(climate_variable) %>%
+   summarise(max_val = max(value, na.rm = TRUE),
+             range_val = max(value, na.rm = TRUE) - min(value, na.rm = TRUE))
> annotation_df <- data.frame(
+   climate_variable = climate_vars,
+   x = 1.5,
+   y = ranges$max_val + 0.1 * ranges$range_val
+ )
> anno_rmean <- annotation_df %>% filter(climate_variable == "rmean")
> anno_tmean <- annotation_df %>% filter(climate_variable == "tmean")
> anno_tmaxsum <- annotation_df %>% filter(climate_variable == "tmaxsum")
> anno_NDMI16 <- annotation_df %>% filter(climate_variable == "NDMI16")
# Create plot for rmean
> p_rmean <- ggplot(data_rmean, aes(x = structure, y = value)) +
+   geom_boxplot(aes(fill = structure),
+                outlier.shape = NA,
+                width = 0.6,
+                alpha = 0.8) +
+   geom_jitter(aes(color = haplotype, shape = haplotype),
+               width = 0.1,
+               alpha = 0.8,
+               size = 2.5) +
+   geom_text(data = anno_rmean,
+             aes(x = x, y = y, label = ""),
+             size = 6) +
+   scale_fill_manual(values = c("P1" = "#ffb300", "P2" = "#00796b")) +
+   scale_color_manual(values = c("H1" = "#ff0000", "H2" = "#00b0f0")) +
+   scale_shape_manual(values = c("H1" = 1, "H2" = 10)) +
+   labs(
+     x = "",
+     y = "",
+     color = "",
+     shape = ""
+   ) +
+   theme_minimal(base_size = 14)
# Create plot for tmean with y-axis fixed to 10-25°C
```

```
> p_tmean <- ggplot(data_tmean, aes(x = structure, y = value)) +
+   geom_boxplot(aes(fill = structure),
+                outlier.shape = NA,
+                width = 0.6,
+                alpha = 0.8) +
+   geom_jitter(aes(color = haplotype, shape = haplotype),
+               width = 0.1,
+               alpha = 0.8,
+               size = 2.5) +
+   geom_text(data = anno_tmean,
+             aes(x = x, y = y, label = ""),
+             size = 6) +
+   scale_fill_manual(values = c("P1" = "#ffb300", "P2" = "#00796b")) +
+   scale_color_manual(values = c("H1" = "#ff0000", "H2" = "#00b0f0")) +
+   scale_shape_manual(values = c("H1" = 1, "H2" = 10)) +
+   scale_y_continuous(limits = c(10, 22), breaks = seq(10, 22, by = 5)) +
+   labs(
+     x = "",
+     y = "",
+     color = "",
+     shape = ""
+   ) +
+   theme_minimal(base_size = 14)
# Create plot for tmaxsum
> p_tmaxsum <- ggplot(data_tmaxsum, aes(x = structure, y = value)) +
+   geom_boxplot(aes(fill = structure),
+                outlier.shape = NA,
+                width = 0.6,
+                alpha = 0.8) +
+   geom_jitter(aes(color = haplotype, shape = haplotype),
+               width = 0.1,
+               alpha = 0.8,
+               size = 2.5) +
+   geom_text(data = anno_tmaxsum,
+             aes(x = x, y = y, label = ""),
+             size = 6) +
+   scale_fill_manual(values = c("P1" = "#ffb300", "P2" = "#00796b")) +
+   scale_color_manual(values = c("H1" = "#ff0000", "H2" = "#00b0f0")) +
+   scale_shape_manual(values = c("H1" = 1, "H2" = 10)) +
+   labs(
+     x = "",
+     y = "",
+     color = "",
+     shape = ""
+   ) +
+   theme_minimal(base_size = 14)
# Create plot for NDMI16
> p_NDMI16 <- ggplot(data_NDMI16, aes(x = structure, y = value)) +
+   geom_boxplot(aes(fill = structure),
+                outlier.shape = NA,
+                width = 0.6,
+                alpha = 0.8) +
+   geom_jitter(aes(color = haplotype, shape = haplotype),
+               width = 0.1,
+               alpha = 0.8,
+               size = 2.5) +
+   geom_text(data = anno_NDMI16,
+             aes(x = x, y = y, label = ""),
+             size = 6) +
+   scale_fill_manual(values = c("P1" = "#ffb300", "P2" = "#00796b")) +
+   scale_color_manual(values = c("H1" = "#ff0000", "H2" = "#00b0f0")) +
+   scale_shape_manual(values = c("H1" = 1, "H2" = 10)) +
+   labs(
+     x = "",
+     y = "",
+     color = "",
```

```
+     shape = ""
+   ) +
+   theme_minimal(base_size = 14)
> # Combine plots in a 2x2 grid using patchwork
> combined_plot <- (p_rmean + p_NDMI16) / (p_tmaxsum + p_tmean)
> # Display the combined plot
> print(combined_plot)
```

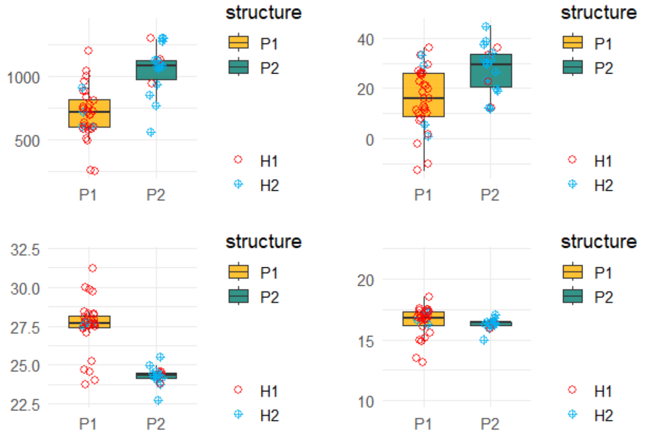

Supplement: Supplementary file 1 — Appendix S1: mec70217‐sup‐0001‐AppendixS1.zip. [file MEC-35-e70217-s001.zip › 3_SCAM_hierarchical_variables_climatic_comparisons_Script_and_process.pdf]
