# Supplementary figures and images for: Genomic Evidence for Dual Introductions, Limited Gene Flow and Niche Preferences in the Invasive Wasp Vespula germanica in South Africa
Source: Mol Ecol. 2025 Dec 22;35(1):e70217. doi: 10.1111/mec.70217 (PMC12745851; doi:10.1111/mec.70217)

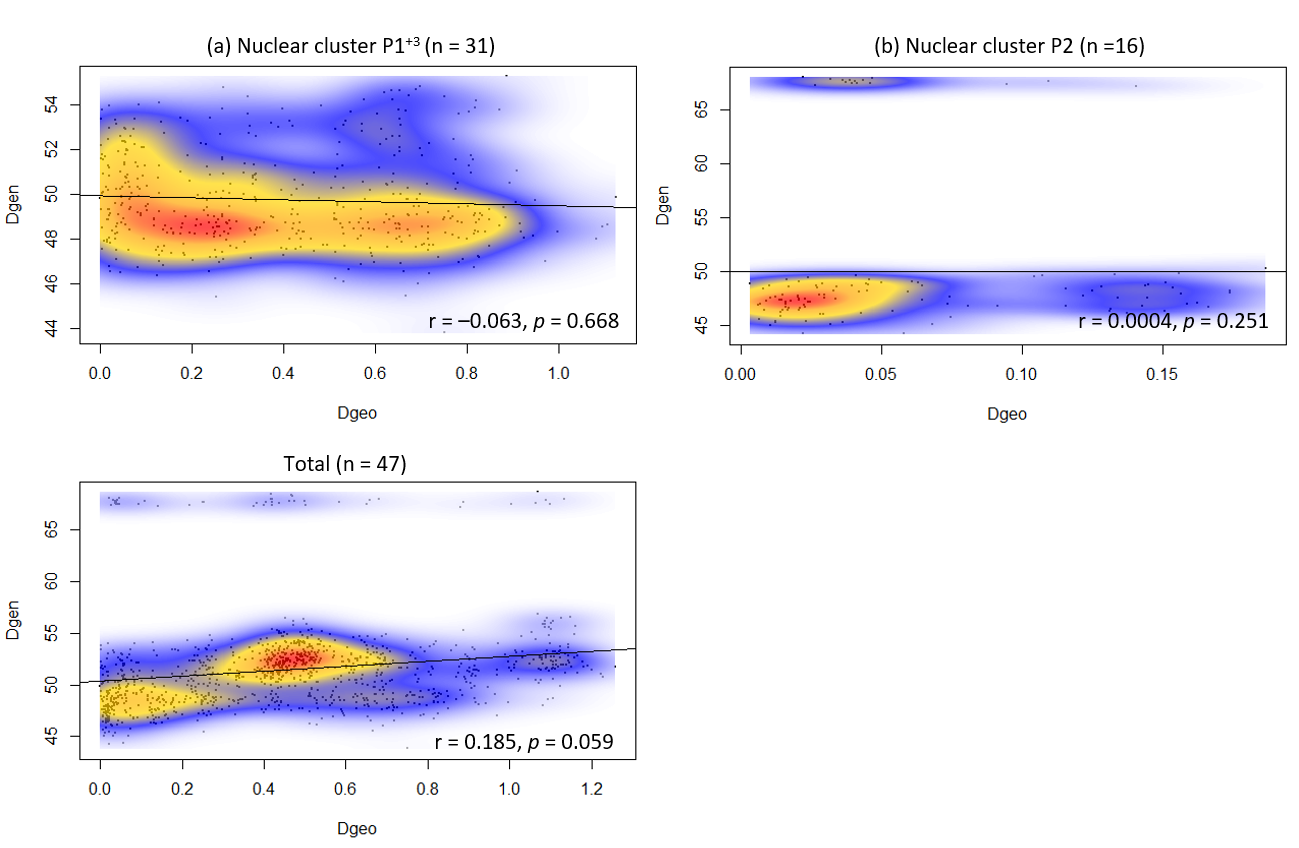

Supplement: Supplementary file 1 — Appendix S1: mec70217‐sup‐0001‐AppendixS1.zip. [file MEC-35-e70217-s001.zip › FigureS3_mantel_IBD.tif]

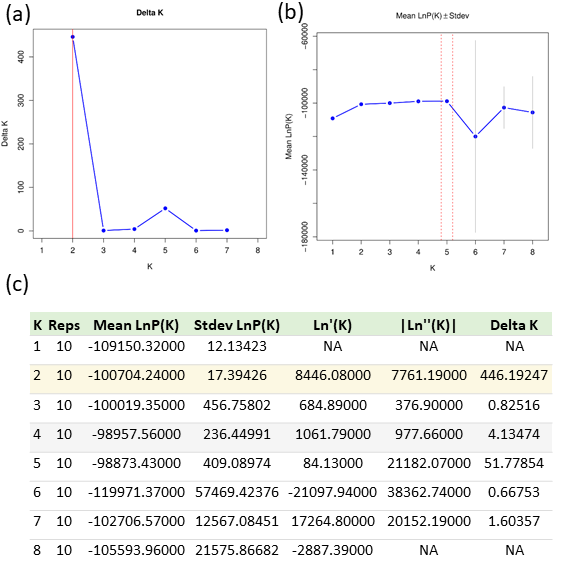

Supplement: Supplementary file 1 — Appendix S1: mec70217‐sup‐0001‐AppendixS1.zip. [file MEC-35-e70217-s001.zip › FigureS1_structure_results.tif]

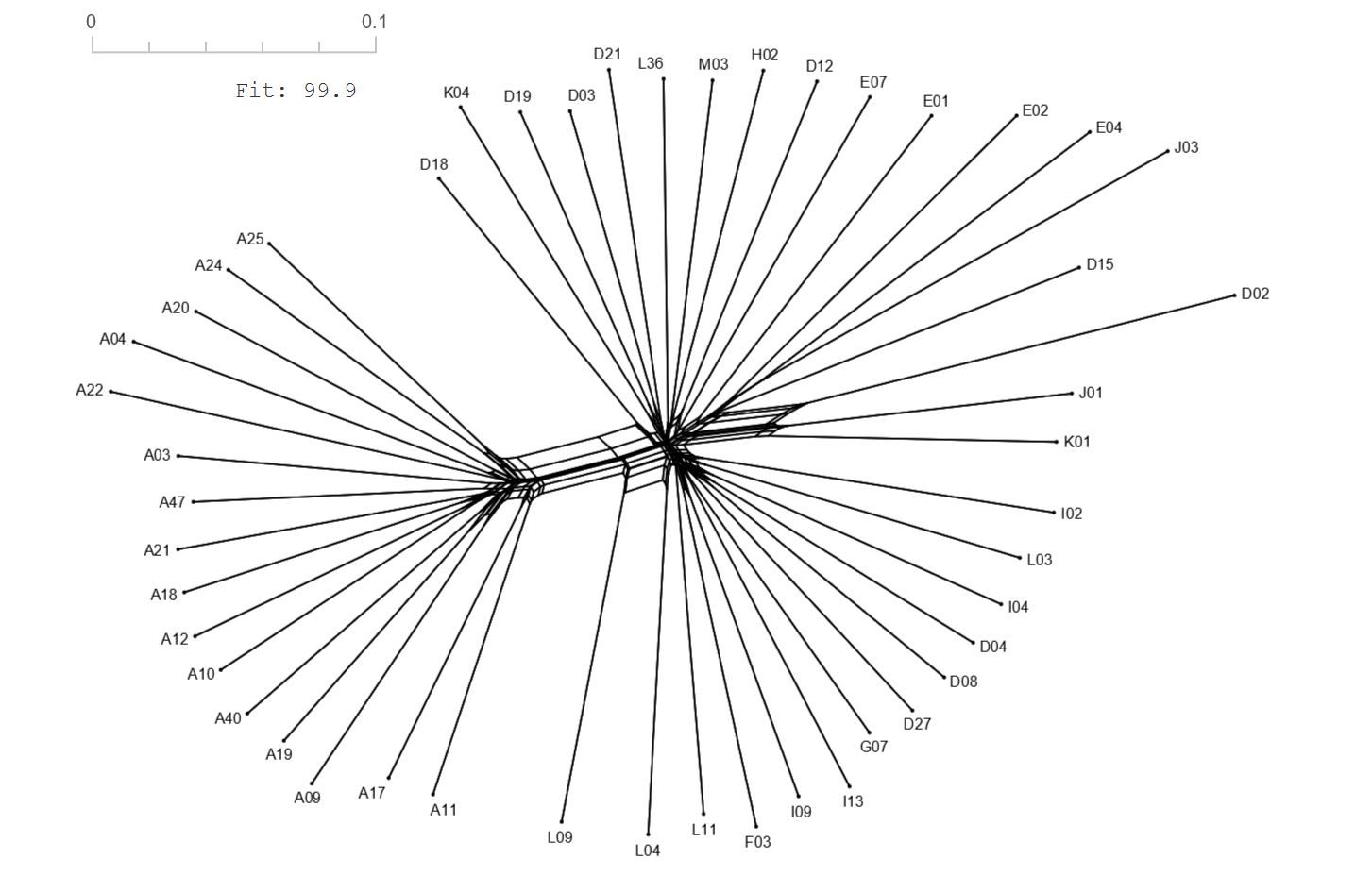

Supplement: Supplementary file 1 — Appendix S1: mec70217‐sup‐0001‐AppendixS1.zip. [file MEC-35-e70217-s001.zip › FigureS2_tree_average_states.tif]
